# Examining the potential of type V DESs for the solvent extraction of metal ions†

**DOI:** 10.1039/d5gc00489f

**Published:** 2025-03-25

**Authors:** Nicolas Schaeffer, Inês C. M. Vaz, Maísa Saldanha Pinheiro, Felipe Olea, Takafumi Hanada, Sandrine Dourdain, João A. P. Coutinho

**Affiliations:** a CICECO – Aveiro Institute of Materials, Department of Chemistry, University of Aveiro 3810-193 Aveiro Portugal nicolas.sch@ua.pt; b Laboratory of Separation Process Intensification (SPI), Department of Chemical Engineering and Bioprocess, University of Santiago de Chile (USACH) Chile; c Department of Applied Chemistry, Graduate School of Technology, Industrial and Social Sciences, Tokushima University 2-1 Minamijosanjima Tokushima Japan; d Institut de Chimie Séparative de Marcoule, ICSM, CEA, CNRS, ENSCM, Univ Montpellier BP 17171 Marcoule 30207 Bagnols-sur-Cèze France

## Abstract

Growing interest in sustainable and efficient metal ion separation has led to the exploration of non-ionic deep eutectic solvents (DESs), also known as type V DESs, as promising alternatives to conventional organic phases in solvent extraction (SX). This work summarizes recent developments, focusing solely on the use of non-ionic DESs and excluding ionic DESs, for the separation of metal ions from synthetic and real leachates. The review does not aim to exhaustively cover all studies but focuses on the molecular mechanisms of SX, how inherent properties of DESs influence these mechanisms, and how they can be harnessed to improve the separation selectivity. It further highlights the physico-chemical properties of DESs in SX and compares them with traditional systems, emphasizing similarities and new opportunities. The overall aim is to clarify the potential and limitations of type V DESs in SX, including their often touted credentials as “green solvents”, and to offer guidelines for their practical use and addressing skepticism towards novel solvents in hydrometallurgy.

## Abstract 2

The potential of hydrophobic type V DESs for the liquid–liquid separation of metal ions is reviewed along with the properties relevant to their application and their potential advantages and limitations compared to conventional solvent extraction.

## Graphical abstract

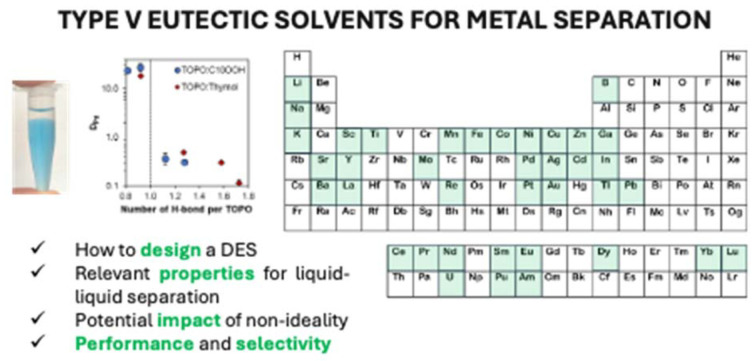

**Green foundation**

1. Non-ionic eutectic solvents, also known as type V DESs, are increasingly proposed as alternatives to conventional organic phases in solvent extraction. This work critically reviews the mechanisms of metal extraction in these novel solvents, their properties and their potential advantages and limitations.

2. Type V DESs should be considered as an *evolution* rather than *revolution* of organic solvent extraction phases, which could lower the barriers to industrial implementation if demonstrable improvements are achieved. However, there is a lack of research on the green credentials of these solvents.

3. Hydrophobic DESs are not a universal solution to all metal separation challenges, but, when effectively utilized, have the potential to offer an additional approach for addressing both current and future solvent extraction issues.

## 1. Introduction

In the face of growing anthropogenic environmental pressures, societies are re-evaluating the existing linear consumption model of “take–make–dispose” and shifting towards a more holistic circular economy framework. Despite a growing global consciousness, the demand for mineral fuels, metal ores, and industrial minerals significantly expanded from 11.3 billion tonnes in 2000 to 17.3 billion tonnes in 2020.^1^ This represents a rapid growth of 52% in two decades that presents no signs of slowing down. Metal ores are forecast to be among the fastest growing primary commodities in the next decades driven in large parts by the expanding electrification and digitalisation of modern society as well as the transition to green sources of energy.^2^ Despite the large uncertainty associated with future predictions based on the stringency of various climate scenarios, the demand for metals from clean energy technologies by 2040, relative to 2020 levels, is conservatively estimated to increase by 1.7× for Cu, 2.0× for Mo, 3.0× for Mn, 3.4× for rare earth elements (REEs), 6.0× for Co and Ni, and 13× for Li.^3,4^ The need for greater production stands in stark contrast to the declining ore grade of most metals, translating into the requirement for greater amounts of energy, water and chemicals to extract the same resource.^5^ This is particularly concerning as mineral and metal production currently accounts for approximately 10% of global energy-related greenhouse gas emissions due the energy-intensive nature of its operations.^6^ At the other end of the production chain, the global amount of electronic goods placed on the market and of e-waste generated in 2022 alone was a staggering 96 and 62 million tonnes respectively, with the latter expected to reach 82 million tonnes by 2030.^7^ The inherent value in 2022 of the metals embedded in e-waste was $91 billion with only $28 billion of this value reclaimed by recycling efforts. Furthermore, most of this $28 billion is associated with base metal recycling, primarily Fe and to a lesser extent Al, Cu, and Zn, with some contributions from precious metal recovery. Unfortunately, the recycling of specialty metals present in minor concentrations remains negligible in most cases, below 1% for REEs for example, suggesting that significant work is still required to achieve a fully circular economy.^7^

Now more than ever sustainable metallurgy and innovative separation processes are required to address the need to recover more from progressively more dilute and heterogeneous matrices. Whilst pyrometallurgy (relying on heat) is generally employed for the treatment of sulphide minerals and more concentrated “urban ores”, hydrometallurgy (relying on chemicals) is increasingly favoured by industry for its ability to recover metals from low-grade mixed-metal sources, with a smaller footprint and lower emissions, and aiming at the potential recycling of chemical reagents.^8^ The shift towards hydrometallurgical operations is best illustrated by the battery recycling industry, with established battery recycling operations depending on pyrometallurgy whilst most of the planned battery recycling capacity relies on either hydrometallurgy or a mixture of pyro- and hydrometallurgy.^4^ Since the days of Jabir Ibn Hayyan over a millennium ago, the hydrometallurgical process can be summarized by three main operations: leaching involving the transfer of metals from a solid matrix to an aqueous medium, followed by metal separation to recover and concentrate the ion of interest, and finally refining to regenerate a solid metal sample. The growing diversity and heterogeneity of primary and urban ores, combined with the need for increasingly pure reagents for the manufacture of advanced materials, is placing renewed pressure on separation technologies.

Solvent extraction (SX) is the major industrial process used to separate and purify target metals from complex leachates through their liquid–liquid phase transfer to an immiscible organic phase.^8^ This is achieved by means of amphiphilic molecules (extractants) with a chelating polar group to ensure metal coordination and a hydrophobic part providing solubility in the apolar phase (diluent). However, SX can suffer from a complex process flowsheet arising from the poor separation selectivity between ions of similar charge and ionic radius. For example, the stable oxidation state of +3 and near-identical chemistries of most REEs make their mutual separation both technologically and environmentally challenging. Identified as one of the key separation challenges of the 21^st^ century,^9^ industrial REE separation can occur over a hundred counter-current extraction stages, generating substantial volumes of volatile, and potentially harmful, organic effluents due to the relatively dilute operating conditions applied, from ppm levels to approximately 15 g L^−1^ of target solute. As such, SX is conservatively estimated to account for 30% of the total associated environmental impacts of RE oxide (REO) production,^10–12^ with a global warming contribution of 35.4 to 72.8 kg CO_2,eq_ per kg of Nd_2_O_3_ depending on the complexity of the ore type as determined by life cycle analysis (LCA).^10^ Assuming a similar order of global warming contributions for the other REOs and considering the estimated total global production of 350 000 tons of REO equivalents in 2023, with the majority separated by SX,^13^ the SX processing of REEs alone generated over 10 000 tons of CO_2,eq_ in the same year. This is, of course, an extremely broad estimate but serves to illustrate that significant opportunities remain to improve the selectivity for target ions over a smaller number, ideally one, of extraction and stripping steps.

It is in this context of increasing technical and environmental constraints that hydrophobic eutectic solvents (ESs) were first proposed as a substitute for the traditional organic phase in SX.^14^ Unlike most pure solvents, ESs are liquid mixtures formed from solid components through solid–liquid equilibrium (SLE), resulting in significant melting point depressions. The properties of the resulting mixture can be customized by selecting different precursors and compositions. Deep eutectic solvents (DESs), a subset of ESs, show significant negative deviations from ideal thermodynamics due to strong intermolecular interactions, causing deeper melting point depressions.^15,16^ DESs are typically made by combining hydrogen bond donors (HBDs) with acceptors (HBAs) and classified into five types (I–V), as described in Table 1.^15,17^ The recently proposed type V DESs use non-ionic precursors,^17^ offering greater flexibility for designing hydrophobic DESs compared to ionic-based types I–IV. Through the incorporation of non-ionic metal extractants as DES components, the resulting hydrophobic phases were applied with promising results for the SX of uranyl, palladium, platinum, gallium, indium, arsenic, lithium, iron or copper.^18–24^ Despite their long-standing application in the pharmaceutical field for example, the application of type V DESs in SX is a young but promising field of study requiring further formalisation.

**Table 1:** Classification of DES types ([C] – organic cation; [X] – halide anion; M – inorganic cation; [C_2_C_1_im]Cl-1-ethyl-3-methylimidazolium chloride; [Chol]Cl – choline chloride; TOPO – trioctylphosphine oxide)

Type	Description	General formula	Example	Ref.
I	Organic salt + metal halide	[C][X] + MX_*y*_	[C_2_C_1_im]Cl + AlCl_3_	25
II	Organic salt + metal halide hydrate	[C][X] + MX_*y*_·*z*H_2_O	[Chol]Cl + CrCl_3_·6H_2_O	26
III	Organic salt + hydrogen bond donor	[C][X] + HBD	[Chol]Cl + urea	27
IV	Metal salt hydrate + hydrogen bond donor	MX_*y*_·*z*H_2_O + HBD	Ce(NO_3_)_3_·6H_2_O + urea	28
V	Hydrogen bond donor + hydrogen bond acceptor	HBD + HBA	Phenol + TOPO	19

This review aims to summarise recent developments on the use of non-ionic hydrophobic ESs for the hydrometallurgical SX separation of metals from both synthetic and real leachates, with all data used in the figures available, when possible, in the ESI.† Applications using ionic DESs and/or organic solutes are not considered as part of this work due the different mechanisms controlling the partition in these systems, with the reader redirected to other recent reviews on these topics.^29–33^ Apart from a limited number of more technical paragraphs, this work is designed for a general audience. To avoid burdening the text with large tables, collected data presented in figures are available in the ESI.† Importantly, this review does not claim nor strive to provide an exhaustive overview of all works to date, focusing instead on the underlying molecular mechanisms behind extraction and how the non-ideality of the eutectic phase influences the latter. This is complemented by summarising the intrinsic properties of DESs that provide them with physico-chemical advantages for SX. A constant theme throughout is a comparison of the DES structure and SX tendencies with the corresponding classical SX system, drawing inspiration from systems containing mixed extractants or phase modifiers and their hydrogen bond interactions. When insufficient literature is available, it is worth noting that certain discussion topics on DES behaviour are based on extrapolation from a comparable system in classical SX due to the greater maturity of the field. By accentuating the parallels between industrially well-known and applied SX systems and type V DESs, this work aspires to demystify the use of type V DESs, identify their limitations to provide guidelines for their advantageous application, and demonstrate their practicality to avoid the scepticism often addressed towards the use of novel solvents (ionic liquids and DESs) in hydrometallurgy.^34^

The review is divided into three parts. Firstly, to better define the aim and scope of this work as well as to avoid common fallacies regarding the “special” nature of eutectic systems, a short definition of what constitutes a DES is discussed. This is followed by a summary of the forces driving SX, including hydrogen bonding, and how these changes can be harnessed in DESs. Secondly, a comparison of type V DES properties relevant to liquid–liquid separations, namely liquid phase structuration, hydrodynamic properties, solvent parameters, and stability and environmental impact, are compared against those of conventional organic phases in SX. Finally, a conclusion and future perspectives are provided. As will be shown, hydrophobic DESs are by no means the answer to all SX issues as the choice of system depends on a myriad of parameters including the nature of the solute and contaminants, their speciation, and the composition of the aqueous and organic phases. However, if properly harnessed, they could provide an additional avenue to tackle some of the current and future SX challenges.

## 2. DESs and solvent extraction – a useful complication?

### 2.1. Non-ideality and deep eutectic solvents

**Fig. 1:**
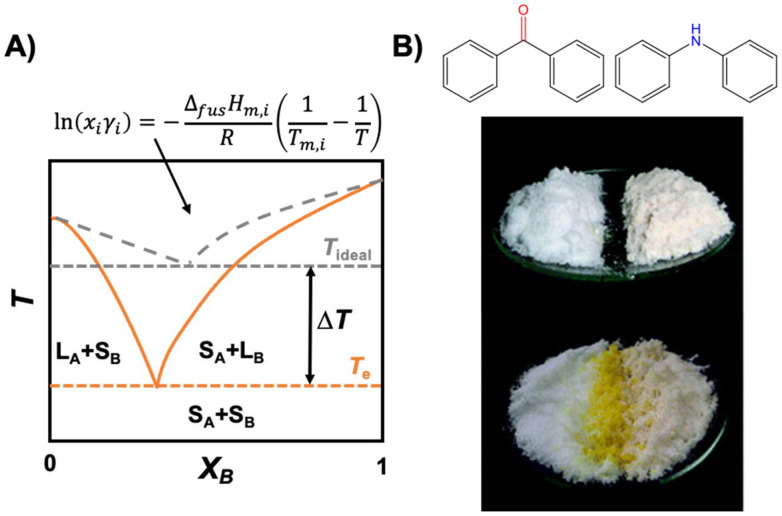
(A) Temperature (*T*)–composition (molar fraction *x*) phase diagrams of A + B in which the ideal and real solid–liquid phase boundaries are represented by dashed grey and solid orange lines, respectively. *T*_ideal_ and *T*_e_ are the ideal and real eutectic temperatures, whilst *S*_*i*_ and *L*_*i*_ indicate the solid and liquid states of component *i*. (B) Photograph of benzophenone (left) and diphenylamine (right) and the resulting yellow eutectic solvent formed along the interface. This figure has been adapted from ref. 37 with permission from the Royal Society of Chemistry, copyright 2007.

Consider the hypothetical temperature *vs.* composition phase diagram of components A and B in Fig. 1A, which upon mixing form a solution AB. According to the Gibbs phase rule, an invariant point exists in temperature/composition space when three phases—A(s), B(s), and AB(l)—are in equilibrium, corresponding to the intersection of the liquidus curves for A and B, and marks the composition with the lowest melting temperature (*T*_e_). Under constant temperature and pressure, the chemical potential of component *i* is given by eqn (1):1
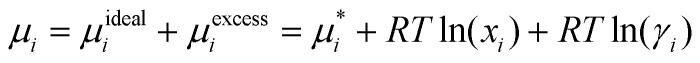
where *T* is the temperature, *R* is the gas constant, and *γ*_*i*_ is the activity coefficient of component *i* to account for deviations from ideality. At the SLE, the chemical potential, *μ*_*i*_(s), of component *i* in the solid phase must equal the chemical potential in the liquid phase, *μ*_*i*_(s) = *μ*_*i*_(l). Furthermore, the chemical potential difference, 
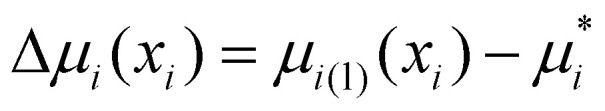
, of component *i* in the liquid mixture (*μ*_*i*(l)_) and in the pure liquid 
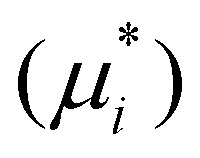
 is related to the melting enthalpy, Δ_m_*H*_*i*_, melting point *T*_m,*i*_ of the pure component, and the melting point of the mixture, *T*. From this and assuming pure solid phases and neglecting the temperature influence on the heat capacities, classical thermodynamics proposes eqn (2) to describe these melting curves:^35^2
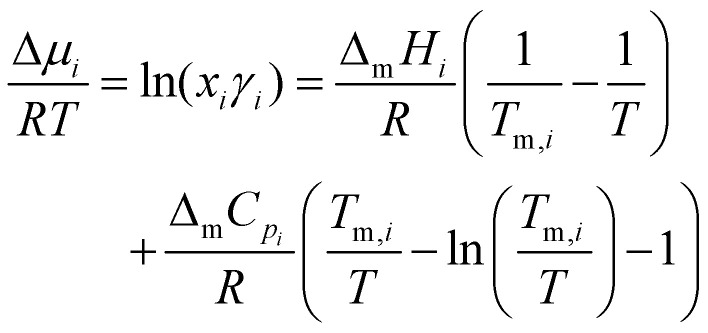
where Δ_m_*C*_p_ is the difference between the molar heat capacity of compound *i* in the liquid and solid phases. For typical type V eutectic systems for which the equilibrium temperature is less than 100 K below the melting temperature of the pure compounds, the last term can be neglected to yield well-known eqn (3):^36^3
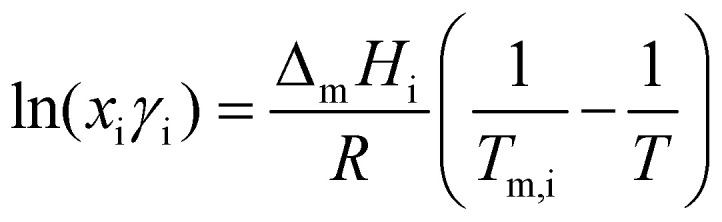


From these equations several considerations can be derived regarding what to expect and not expect from a “deep” eutectic solvent. Beyond thermodynamics, practicality imposes a further condition for eutectic solvents, namely that these should be liquid at operating temperatures suitable for SX applications. A visual example of the liquefaction potential afforded by component inclusion in a eutectic mixture is presented in Fig. 1B, showing spontaneous liquefaction (in yellow) at the interface of the benzophenone and diphenylamine solid phases.

(1) For a solution in which intermolecular interactions are equal to those in the pure compounds (AB = AA = BB)

In an ideal mixture, *μ*^excess^_*i*_ = 0 and *γ*_*i*_ = 1 (dashed grey line in Fig. 1A), such that the decrease of the solution chemical potential, and therefore its melting point, is solely due to the mixing entropy and the maximisation of local disorder, and is proportional to the molar fraction (*x*_*i*_).^38^ The freezing point depression of a mixture (that does not form a solid solution) relative to that of the pure compounds is not in itself an indication of a “deep” eutectic solvent and there are no special magic intermolecular interactions that define the eutectic composition relative to others.^39^ The most robust evaluation of a system is indeed that a DES or a simple eutectic solvent remains through the determination of the SLE phase diagram.

(2) For a solution in which intermolecular interactions are greater than those in the pure compounds (AB ≥ AA or BB)

The extent of the melting point depression and therefore the compositional width of the liquidus region is dictated by the negative deviations from ideal mixing (*γ*_i_ < 1, full orange line in Fig. 1A). This is typically assigned to dominant favourable enthalpic interactions between the mixture's components through any type of strong interaction, usually, but not restricted to, hydrogen bonding.^40,41^ However, the presence of hydrogen bonding in a mixture is not a sufficient condition for being considered a DES, as shown for ideal mixtures of carboxylic acids.^42^ On the other side of the spectrum, systems akin to protic ionic liquids in which partial or complete proton transfer occurs due to a significant p*K*_a_ difference between the constituents, as observed for example in mixtures of lidocaine and fatty acids,^43^ are also not considered due to the formation of new chemical species. Finally, although the mixing entropy is typically considered as ideal and neglected in the description of non-ideality, this is a simplification. In the *tert*-butyl alcohol (TBH) + perfluoro-*tert*-butyl alcohol (TBF) mixture, which presents a negative excess Gibbs energy of mixing, the highly negative excess enthalpy is partially quenched by the negative excess entropy.^41,44^ The directionality of the hydrogen bond pair is enthalpically favourable but comes with an entropic penalty. An additional situation for which the excess mixing entropy should most likely be considered is when the eutectic components significantly differ in molar volumes.^45^ Unfortunately, there are few works reporting excess properties of DESs to appreciate the contribution of entropy relative to the dominant enthalpic contribution.

**Fig. 2:**
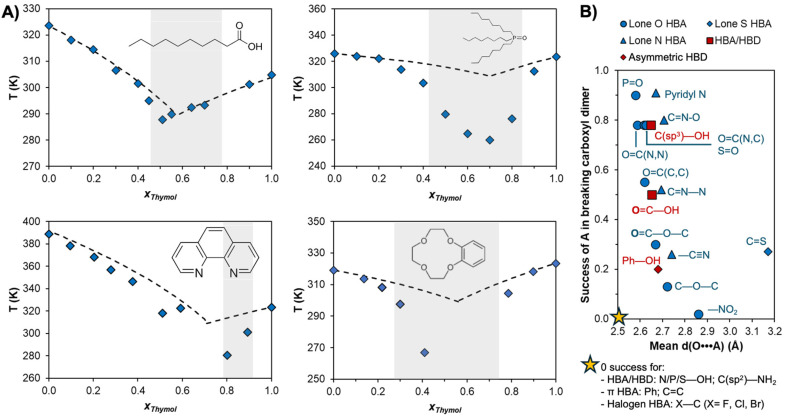
(A) Temperature–composition phase diagrams of thymol with, in clockwise order, decanoic acid,^46^ TOPO,^47^ benzo-12-crown-4 ether (unpublished, data in Table S1 of the ESI†), and 1,10-phenanthroline.^48^ Experimental and ideal solid–liquid phase boundaries are represented by blue diamonds and dashed lines respectively, and the liquid range at 298 K is highlighted in grey. (B) Probability of the relative success of hydrogen bond acceptors competing for the carboxylic acid donor against the average hydrogen bond length in crystals (data taken from ref. 52).

To illustrate the above discussion and its application to solvent extraction, the SLE phase diagrams of thymol (a natural monoterpenoid phenol) as a HBD with common metal extractants including decanoic acid,^46^ TOPO,^47^ 1,10-phenanthroline,^48^ and benzo-12-crown-4 ether are presented in Fig. 2A. The greater acidity of the phenolic hydrogen makes thymol an excellent HBD but poor HBA, with weaker hydrogen bonds than comparable alcohols in the liquid state.^49^ As illustrated in Fig. 2A, mixing of thymol with lone HBAs presenting limited to no self-interactions *via* hydrogen bonding, such as phosphine oxides and glymes, results in extensive negative deviations from non-ideality as AB > AA. In contrast, the stability of the carboxylic dimer (AB = AA) results in a quasi-ideal phase diagram despite the greater number of overall hydrogen bonding moieties in the mixture relative to the TOPO system. This simple comparison emphasises that the presence of hydrogen bonding is secondary to the “right type” of hydrogen bonding to promote significant non-ideality.

The phase diagram of thymol + 1,10-phenanthroline presents more complex behaviour than the others in Fig. 2A, as characterised by two eutectic points at approximately *x*_thymol_ ∼ 0.7 and 0.5, and the formation of a co-crystal at *x*_thymol_ ∼ 0.6.^48^ Favourable intermolecular interactions, particularly directional ones like hydrogen bonding, must be balanced with stereochemical hindrance that might otherwise give rise to co-crystal formation and the accompanying rise of the melting temperature at that ratio. Taking a leaf out of the “anti-crystal engineering” design rules for ionic liquids,^50^ it appears that components with functional groups that hinder efficient packing (conformational flexibility, the number and accessibility of interaction sites, the presence and nature of alkyl chain substituents, *etc*.) limit the occurrence and impact of co-crystals on the SLE. For example, the substitution of thymol by butylated hydroxytoluene, which possesses a phenolic OH group sandwiched between two *tert*-butyl groups, when paired with 1,10-phenathroline yielded a ∼60 K relative increase in the melting point due to the formation of a strong 1 : 1 co-crystal.^48^ Another example of this is in the prototypical thymol + menthol mixture, which displays strong deviations from ideality as well as co-crystals at molar ratios of 3 : 1 and 3 : 2, and a solid solution region slightly above the 3 : 1 molar ratio.^51^

In addition to chemical intuition based on the generalities provided, existing experimental data from various chemical fields as well as modelling tools are available to guide the design of non-ionic DESs. For example, on the experimental side, Fig. 2B shows the relative success of hydrogen bond acceptors containing a given functional group of breaking the strong carboxylic acid dimer in crystals from a large database study.^52^ For the lone HBAs, the probability approximately decreases with decreasing basicity of the receptor group, granting a quick qualitative ranking of potential HBA functional groups for designing non-ideal eutectic mixtures.

On the modelling side, several families of approaches are available for the *in silico* screening and/or prediction of SLE phase diagrams, namely excess Gibbs energy models, equations of state, computational simulations, and machine learning. For a more detailed description of each approach, the reader is directed to dedicated reviews.^53,54^ By far the most employed is the excess Gibbs energy approach due to the popularity of COSMO-based models and its ability to bridge the molecular and macroscopic thermodynamic levels. This is a predictive model to describe the SLE that does not require experimental input beyond the melting properties of the pure components. As validation, Teixeira and co-workers^55^ showed that COSMO-RS could estimate the melting temperature of 133 different binary type V eutectic systems with an average absolute deviation of 7.4 K, comparable to the UNIFAC model without the need to derive group interaction parameters. The predictive capacity of the model can be further improved by fine-tuning the conformer selection of the DES components by using the model in a semi-predictive manner.^56^ Of note is the integration of COSMO generated Sigma profiles as *in silico* inputs for machine learning models for the prediction of melting properties and phase diagrams.^57,58^ Active learning models outperformed common thermodynamic models such as the nonrandom two-liquid (NRTL) model with fewer experimentally required training points.^58^

**Fig. 3:**
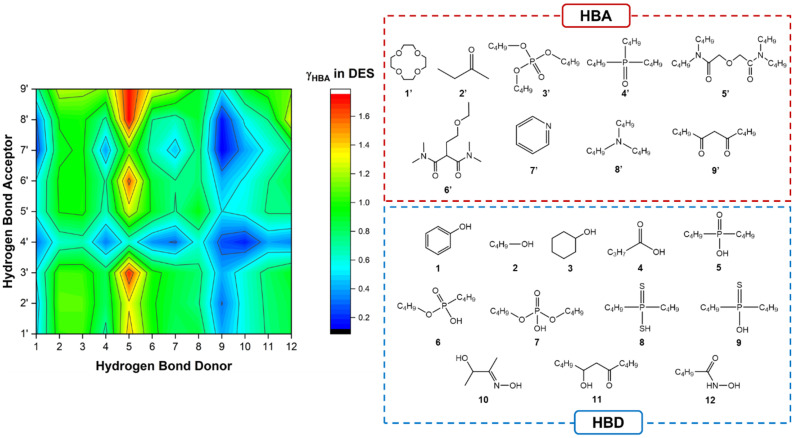
The activity coefficients at infinite dilution of the HBA (ln *γ*_HBA_) in HBA–HBD eutectic mixtures at *x*_HBA_ = 0.5. All results are available in Table S2 of the ESI.†

The ability to establish metal–ligand interactions is conferred by the presence of chelating moieties (*e.g.*, phosphine oxide, diglycolamide, ketone, *etc.*). Such predictive approaches allow to rapidly understand how the incorporation of various metal ligands influences the resulting DES, how to design deviations from thermodynamic ideality and make their properties suitable for SX. This is exemplified by the COSMO-RS screening in Fig. 3, in which model molecules representative of commonly used neutral and acidic extractants are evaluated for their probability of DES formation, as determined by a negative activity coefficient of the HBA in the mixture. Consistent with the previously presented results, phenol and phosphine oxide moieties are identified as the optimal HBD and HBA groups, respectively, showing negative deviations with all tested compounds. In contrast, few HBAs can displace the strong self-association of the phosphinic acid group, making the latter a poor HBD despite its acidity. Interestingly, monothiophosphinic acid (representative of the extractant Cyanex 302®) appears to be an excellent HBD and warrants future characterisation as a DES component. A second noteworthy result is that the number of HBA sites does not appear to contribute to the solvent non-ideality compared to the Lewis basicity of the acceptor group, although more work is required to confirm this. Perhaps the better question is if a truly “deep” eutectic solvent is indeed necessary or advantageous for SX applications; this is explored in sections 2.3 and 2.4.

### 2.2. DES component selection

The criteria for DES component selection can be divided into two categories. The first is transversal to any DES formation and depends on the melting properties of the constituents to obtain a final liquid mixture. Employing phase equilibria conditions, Δ_m_*G* = 0, such that the process is dictated by the difference in enthalpy and entropy between the phases. Rearranging the Gibbs equation provides the following relationship:4
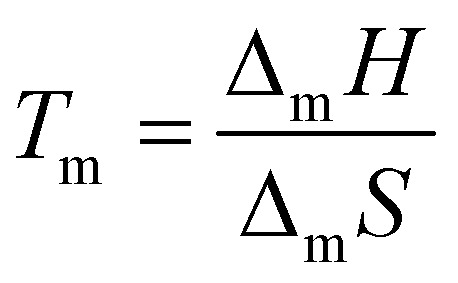


**Fig. 4:**
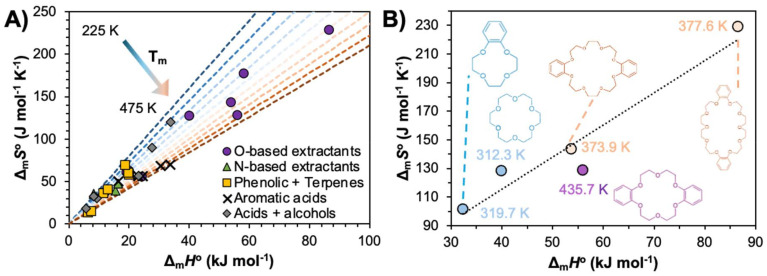
Comparison of Δ_m_*H*_*i*_, Δ_m_*S*_*i*_, and *T*_m,*i*_ for (A) reported components in type V “deep” and ideal eutectic solvents (symbol shape identifies the compound family, and the coloured dashed lines indicate the melting temperature), and (B) crown-ether extractants. All data and respective references are available in Table S3 of the ESI.†

Considering that even strong non-ideality only yields an additional decrease in the DES melting point of 10 to 50 K relative to ideal mixing,^47^ a good starting point for obtaining liquid DES compositions at room temperature comes from the careful selection of components. From eqn (4), it can be deduced that the reduction of the melting point is achievable by minimizing Δ_m_*H*_*i*_, maximizing Δ_m_*S*_*i*_, or a combination of both. The reported melting properties of compounds employed in non-ionic eutectic solvents are presented in Fig. 4A. For a better illustration of how the same properties can be manipulated for a given class of compounds by varying the structure, the melting properties of various crown-ether extractants are shown in Fig. 4B.^59^

Minimizing Δ_m_*H*_*i*_ requires balancing the strength of cohesive forces in both the solid and liquid phases, whilst maximising Δ_m_*S*_*i*_, involves reducing the entropy in the solid state while increasing it in the liquid state. In this sense, although both depend on the same hydrogen bond interactions, DESs are at the opposite end of the conceptual spectrum from hydrogen-bonded frameworks.^60^ Suggesting opposite design criteria for the latter, it appears that liquid state disorder is promoted by molecules with a non-rigid backbone (conformational entropy), a lack of symmetry in the location of donor and acceptor sites, and a mismatch in the number of donor and acceptor sites (configurational entropy). Secondary interactions beyond hydrogen bonding can also increase (π–π-stacking) or decrease (ether groups or alkyl branching) the stability of the solid phase. This is exemplified in Fig. 4A, showing that aromatic acids present generally greater *T*_m_ values than the equivalent *n*-alkyl acid. Increasing the size of the molecule without altering the functional groups, or significantly changing its symmetry and shape, results in a general enthalpy–entropy compensation, as illustrated in Fig. 4B, with only a modest increase in *T*_m_ due to greater dispersive interactions. However, the addition of two symmetrically situated benzene moieties to an 18-crown-6 ether backbone raises *T*_m_ by 123 K. In turn, *T*_m_ can be reduced by 62 K from dibenzo-18-crown-6 ether to dibenzo-24-crown-8 ether by increasing the ether chain length, providing greater flexibility to the structural backbone.^59^

The second criterion is determined by the application and varies based on the desired end-use and the accompanying restrictions. The use of DESs for hydrometallurgical liquid–liquid separation imposes several demands on the design of the solvent and therefore the component selection.^61^ First and foremost, at least one of the DES components should form selective extractable complexes with the metal ion of interest during extraction, whilst being easy to strip and regenerate the solvent. A brief overview of general extraction mechanisms is presented in section 2.4. Fully incinerable components containing only carbon, hydrogen, nitrogen and oxygen atoms should be privileged, as well as those presenting low vapour pressures and a high flash point. If working in an ionizing environment, the components should be resistant towards irradiation and, if not, be regenerable. Due to their SX application and the aggressive and oxidative nature of the aqueous phase, low water solubility and high chemical stability are required. For example, phenolic and alcohol compounds present limited stability in the presence of nitric acid due to oxidation and/or nitration reactions, whilst phenols are known to undergo condensation reactions in the presence of HBAs.^62^ The DES must be a stable liquid, resistant to phase transitions (either third phase formation or solid precipitation) and jellification upon metal loading, as observed in the TOPO + decanoic acid system upon saturation with [PtCl_6_]^2−^.^63^

Although the discussion focuses on chelating and hydrogen bonding functional groups, it is important to note that a nuanced structure–performance relationship exists in SX when designing the apolar fraction of the extractant as this influences steric and electronic effects to coordination as well as extractant packing. For example, subtle changes to the structure around the polar head group of diglycolamide extractants, such as cyclic *vs.* linear alkyl groups, the degree of alkyl chain branching, and structural symmetry, not only result in changes to the lanthanide cation extraction strength but also in a dramatic shift in selectivity along the series.^64^ The same is true for the thermal properties of DESs; as Mannucci and co-workers demonstrated, beyond hydrogen bonding, steric factors also exert a dominant role in the deviation from thermodynamic ideality.^56^

### 2.3. Hydrogen bonding in type V DESs

The importance of hydrogen bonding both as the interaction locus for type V DES formation and its role in SX warrants a short discussion. IUPAC defines a hydrogen bond as:^65^

“*an attractive interaction between a hydrogen atom from a molecule or a molecular fragment X–H in which X is more electronegative than H, and an atom or a group of atoms in the same or a different molecule, in which there is evidence of bond formation*”.

Importantly, the definition lacks any specific criteria to be fulfilled, instead providing several criteria and characteristics based on geometrics, computational (DFT), and spectroscopic (NMR, Raman, and FTIR) properties. The absence of one or more of the criteria does not signify that hydrogen bonding does not exist, particularly as more than a dozen different hydrogen bond types are reported with dissociation energies spanning more than two orders of magnitude. The IUPAC definition is complemented by a number of classifications of hydrogen bond types based on specific property cut-offs; the most popular proposed by Jeffrey and Steiner are listed in Table 2.^66,67^ As for all things on a spectrum with no natural cut-offs, the values in Table 2 serve as guidelines rather than absolute values, keeping in mind that the proposed values are based on the analysis of traditional hydrogen bonds in the solid state. Furthermore, intermolecular hydrogen bonding typically goes beyond simple HBD–HBA dimers, to organise into two- or three-dimensional assemblies involving multiple donors and acceptors, forming hydrogen bonded chain networks and higher order clusters (trimers, tetramers, and so on). Such hydrogen bonded networks can show deviations from pair-wise additivity in hydrogen bond properties due to co-operativity arising from enhanced polarization.^66^

**Table 2:** Strong, moderate, and weak hydrogen bonds (X−H⋯Y) following the classification of Jeffrey^67^

Criteria	Strong	Moderate	Weak
Interaction type	Strongly covalent	Mostly electrostatic	Electrostatic/dispersive
H⋯Y (Å)	1.2–1.5	1.5–2.2	>2.2
Lengthening H⋯Y (Å)	0.08–0.025	0.02–0.08	<0.02
X–H *vs.* H⋯Y	X–H ≈ H⋯Y	X–H < H⋯Y	X–H ≪ H⋯Y
X Y length	2.2–2.5	2.5–3.2	>3.2
Directionality	Strong	Moderate	Weak
Bond angles (°)	170–180	>130	>90
Bond energy (kJ mol^−1^)	63–167	17–63	<17
IR shift (cm^−1^)	25%	10–25%	<10%
^1^H shift (ppm)	14–22	<14	—

As the aforementioned criteria for defining type V DESs preclude proton transfer, covalent and ionic hydrogen bonds (*e.g.* [NH_4_]^+^·Cl^−^) are therefore absent from these solvents thereby placing an upper boundary on the possible energy of hydrogen bonding in type V DESs. The absence of substantial point charges, which decay as a function of the distance (*r*) according to −1/*r* and dominate over long distance correlations, suggests that type V DES structuration is dominated by shorter range interactions such as dipole–dipole (−1/*r*^3^) and London dispersion (−1/*r*^6^). Relative to the ionic type I to IV DESs, type V DESs provide a more flexible platform for tuning the weak forces relevant to the formulation of organic phases in SX.^68^

Unfortunately, few works go beyond FTIR characterisation of eutectic solvents, providing limited details on the energetics of hydrogen bonding, especially their comparison with the starting compounds in the same liquid state. The strongly non-ideal mixtures of TBF with TBH,^41^ as well as thymol with flavone or flavanone,^69^ presented minimum excess enthalpies in the range of −7 to −14 kJ mol^−1^, with an estimated 80% of the total due to the stronger hydrogen bond interactions between unlike molecules.^69^ DFT analysis within the COSMO solvation continuum at 298 K of the thymol–menthol pair in the liquid phase relative to menthol–menthol indicated a more stable interaction by 5.2 kJ mol^−1^.^17^ An identical analysis showed a stabilisation of the betaine–urea pair by 2 kJ mol^−1^ relative to the urea–urea complex.^70^ These examples serve to show that as the deviation from ideality in DESs depends on the presence of favourable intermolecular interactions relative to those present in the pure compounds, strong hydrogen bonding is not a requirement for DES formation particularly if the HBA does not possess any hydrogen bonding capability in its pure form.

**Fig. 5:**
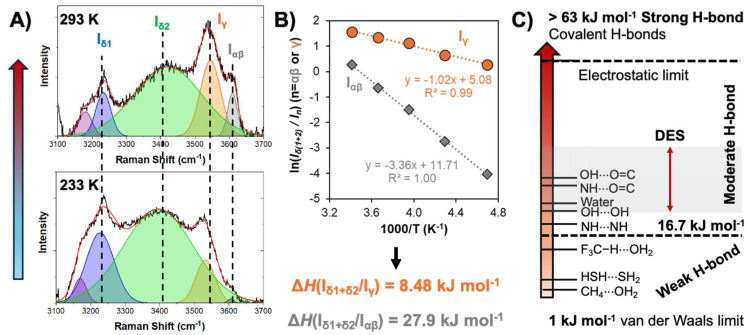
(A) Deconvoluted Raman spectra in the OH stretching region along with band assignments for the liquid thymol + menthol eutectic mixture as a function of temperature for a fixed composition (*x*_thymol_ = 0.5) as a function of temperature. The red line corresponds to the deconvolution fit. This figure has been adapted from ref. 49 with permission from the American Chemical Society, copyright 2021. (B) Logarithmic plot of the ratios *I*_δ(1+2)_/*I*_n_ (n = α, β or γ) *versus* the inverse of the temperature taken from the deconvoluted spectra in panel A between 233 and 293 K. (C) Conceptual scale using the hydrogen-bond classification adapted from Jeffrey^67^ for the potential distribution of intermolecular hydrogen bond enthalpies in DESs.

A final consideration is the temperature dependency of the hydrogen bond and its impact on the non-ideality of the liquid phase. A comparison of the experimental SLE and vapour liquid equilibria (VLE) for the thymol + menthol system at *x*_thymol_ = 0.5 confirmed the drastic reduction in the nonideality of the system with increasing temperature.^49^ The eutectic system is strongly nonideal near the SLE but quasi-ideal at the VLE, with the activity coefficient of thymol in the DES varying from below 0.3 at 223 K to approximately 0.9 at 463 K. The same system was studied by temperature dependent Raman spectroscopy, showing a notable increase in the thymol−menthol hydrogen bonding as the temperature approached the glass transition (∼210 K), as presented in Fig. 5A.^49^ The Raman *ν*(OH) spectrum was deconvoluted in terms of different contributions depending on their involvement in hydrogen bond formation, namely into α and β, γ, and δ OHs.^71–73^ “Free” hydroxyl groups not involved in hydrogen-bonding (α and β; grey in Fig. 5A) as well as hydrogen bonded dimers with lone pairs of the O atom not involved in hydrogen bonds (γ OH, orange) are characteristic of metastable liquid thymol at room temperature and disappear as the temperature decreases in favour of proton accepting and donating δ OHs (blue and green) indicative of DES hydrogen bonded aggregates. Here, we reinterpret these data to extract apparent equilibrium constants, *K*, from the ratios of the Raman area (*I*). Plotting ln(*I*_δ(1+2)_/*I*_n_) (n = α, β or γ) *versus* 1000/*T* results in the linear plot in Fig. 5B, indicating van 't Hoff behaviour. From the slope, estimates for the change in enthalpy of breaking a hydrogen bond from the DES relative to the thymol dimer and free thymol are 8.5 kJ mol^−1^ and 27.9 kJ mol^−1^ respectively. The latter is slightly greater than the typical hydrogen bond energy, in the region of 22 kJ mol^−1^, for water and alcohols.^74,75^

The discussion is summarised in Fig. 5C, in which the probable zone of hydrogen bond energies is highlighted in grey based on the hydrogen-bond classification adapted from Jeffrey.^67^ Type V DESs reported to date primarily rely on alcohols, amines, and carboxylic acid as HBDs, with individual hydrogen bonding enthalpies ranging approximately from 17 to 30 kJ mol^−1^ for the pure HBDs (approximate dimerization enthalpy of 50 to 60 kJ mol^−1^ for carboxylic acids).^76,77^ Considering the criteria for non-ideality being that HBA–HBD interactions are greater than HBD–HBD ones, intermolecular hydrogen bond enthalpies driving DES formation are expected to be in the range of 18 to 35 kJ mol^−1^. The upper bound value is consistent with reported values for DMSO, a lone HBA, with water or formamide,^78,79^ and cannot be significantly exceeded due to the possibility of proton transfer. These of course are guideline values, with multiple possible exceptions and will need readjustment as more data become available.

Relating the pair wise HBA–HBD hydrogen bond strength in DESs to its impact on SX, lessons can be drawn from the existing literature regarding solvent effects and the use of mixed extractants. A direct consequence of increased hydrogen bonding interactions, *i.e.* greater cohesive interactions relative to commonly used aliphatic diluents in SX, is the penalty of cavity formation in the DES phase to accommodate the extracted complex, although this is likely to be partially compensated for by favourable ion complex–DES interactions due to the increased configurational entropy of the extracted ion.^80^ The second is the competitive effect arising from inter-component hydrogen bonding relative to extractant metal complexation. In the pursuit of improved extraction, a common approach is through the use of mixed extractant systems to improve metal ion partition by increasing the lipophilicity of the ion–ligand complex by expanding the solute's coordination sphere and/or by displacement of inner-sphere water molecules.^81,82^ This is typically achieved by the combination of a neutral “solvating” extractant and an acidic one, presenting parallels to the selection criteria for DES formation. In fact, Hanada *et al.* demonstrated this principle in the DES composed of thenoyltrifluoroacetone (HTTA) with trioctylphosphine oxide (TOPO) for lithium extraction.^21^ By the judicious choice of the extractant combination, the resulting distribution ratio can be greater than the sum of extractants when used separately, also referred to as synergistic extraction. However, a given system can demonstrate both synergistic and antagonistic extraction behaviour by simple variations in the molar ratio of the extractants or change in the aqueous phase acidity as demonstrated in mixtures of malonamide with dialkyl phosphoric acid with varying HNO_3_ concentration.^83^ This can be used to increase the selectivity of a separation by suppressing the extraction of an ion relative to the target analyte.

Whilst synergism is challenging to predict solely based on the extractant selection in the absence of metals,^84^ antagonism is often attributed to a competitive relationship between the extractant–ion and the coordination interaction, yielding a reduction of the active or “free” concentrations of the extractants in the organic phase.^81,82^ For example, spectroscopic analysis of a mixture of the HBD bis-(2-ethylhexyl)phosphoric acid (D2EHPA) and HBA octyl(phenyl)-*N*,*N*-diisobutylcarbamoylmethylphosphine oxide (CMPO) in *n*-dodecane shows that the presence of CMPO disrupts the D2HEPA dimer due to the formation of a strong intermolecularly hydrogen bonded adduct (adduct stability constant of log *β* = 3.40). When the D2HEPA concentration exceeds that of CMPO, the resulting D2HEPA–CMPO adduct formation greatly reduces the amount of free CMPO available for metal ion complexation, essentially preventing CMPO from extracting trivalent f-block ions as M(NO_3_)_3_·3CMPO.^85^ Returning to DESs, it therefore appears that a mixture presenting a pronounced non-ideality and strong intermolecular hydrogen bonding could be counter-productive to the overall atomic efficiency of ion extraction as the extractant is both the chelating ligand and DES phase former. Extraction determined by hydrogen bonding of ligands with the outer coordination spheres of metal complexes, such as those typically observed for anionic [PtCl_6_]^2−^,^86^ are likely sensitive to this competitive effect due to the smaller difference between the energies of DES adduct formation and metal complex–ligand binding. It also places the DES molar ratio at the forefront of the variables requiring optimisation as manipulating it can suppress or promote extraction and determine the concentration of free extractant, which must be explicitly considered when attempting to model extraction. In such cases, slope analysis regression is only possible using a complex set of multiple equilibria.

### 2.4. Interactions in SX

The discussion so far might at first glance appear pedantic when discussing solvents for a given application, as one could argue that such nuances between DESs and ESs are irrelevant as long as they work. This section aims to condense the discussion thus far and show not only that this difference does in fact matter for their use in SX but also provides opportunities. The organic phase in SX is best represented as a complex amphiphilic molecular solution presenting ordering over various time and length scales from atomic pair-wise interactions to the formation of mesoscopic colloidal aggregates.^87,88^ The self-assembly of amphiphilic solutes such as metal extractants into three-dimensional structures is driven by weak interactions, namely hydrogen bonding and the hydrophobic effect, and evolves with the nature and concentration of extracted polar solutes.^68^ An extreme example of this evolution in conventional SX is the macroscopic splitting of the organic phase, also called third phase formation, into a diluent-rich and solute-rich phase.^88^

**Fig. 6:**
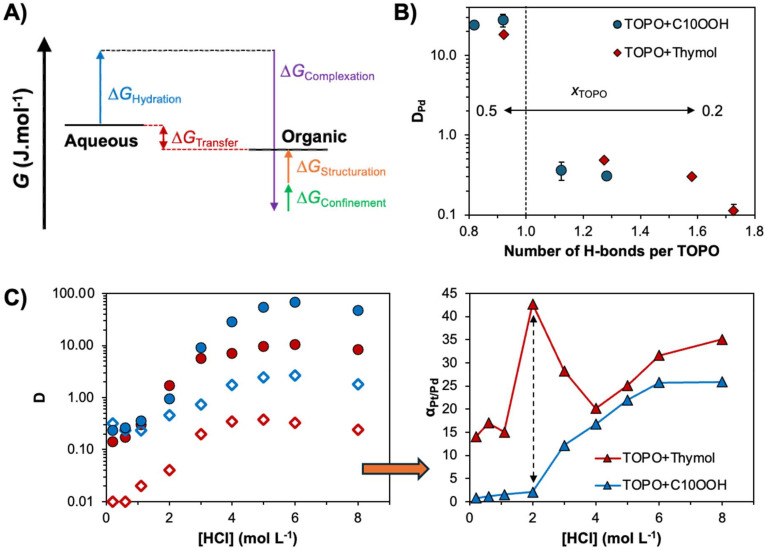
(A) Schematic thermodynamic description of the forces involved in the partition of ionic solute M in SX. (B) Experimental Pd(ii) distribution coefficient in the TOPO + HBD systems (decanoic acid (C_10_OOH) and thymol) as a function of the number of hydrogen bonds per TOPO molecule for varying *x*_TOPO_ from 0.5 to 0.2 (left to right) ([HCl] = 2 mol L^−1^; O/A = 0.5, [Pd] = 2 mmol L^−1^). (C) Pt(iv) (filled symbol) and Pd(ii) (empty symbols) distribution factor and Pt/Pd selectivity (triangle) as a function of HCl concentration and the DES HBD component (*x*_TOPO_ = 0.4, O/A = 0.5, [*M*] = 2.0 mol L^−1^). This figure has been adapted from refs. 47 and 63 with permission from the Royal Society of Chemistry, copyright 2020 and 2021, respectively.

The free energy of transfer (Δ*G*_transfer_) for a given electrolyte (*M*) in SX, schematically illustrated in Fig. 6A, can be written as:^89–91^5
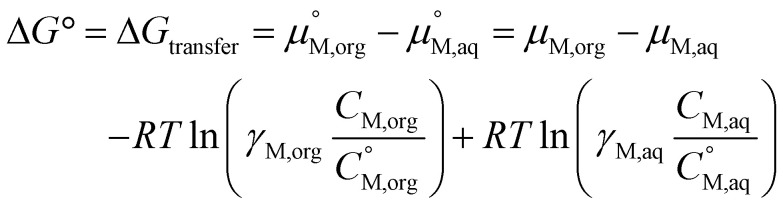
where 
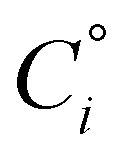
 is the standard concentration in the solvent considered (usually 1 mol L^−1^) and *γ*_*i*_ is the activity coefficient, which tends to 1 when *i* becomes infinitely dilute (asymmetric convention). On this activity scale, the solvent is considered to be an ideal mixture. It is not pure water nor pure diluent since other species, such as the supporting electrolyte, acid, or counter ions and other extracted species in the organic phase, must also be considered. Thus, at equilibrium, and with a defined reference state, Δ*G*_transfer_ can be obtained by the distribution of M between the phases at equilibrium:6
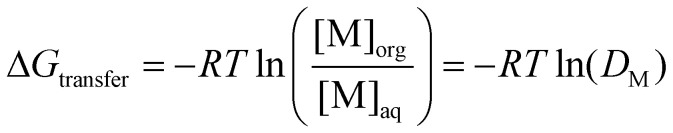
where *D*_M_ is the distribution factor of M. Importantly the standard state of the Gibbs free energy of the transfer standard state is defined for a solvent containing all species apart from the metal salt. It is therefore the free energy per mole of transferred M and not the raw difference of a given sample *versus* a reference state, as classically noted by *μ*° or Δ*G*° in thermochemistry. In eqn (5), all species other than the main one considered are assumed to be part of the solvent and participate in defining the reference state. The separation factor between two ions, M_1_ and M_2_ (*α*_M_1_/M_2__), an indication of selectivity, is given by the ratio of *D*_M_ values and is linked to the transfer energy difference between the two metals, 
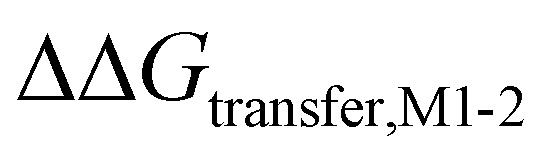
, by:7
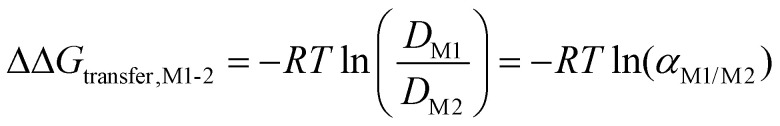


The rationalization of metal transfer from the aqueous to the organic phase requires consideration of the complex molecular events involved, which are often overlooked. When the metal “leaves” the aqueous phase, the commonly discussed dehydration step is not limited to breaking the water–metal interactions; it also involves the “disappearance” of the cavity left by the metal ion, which in turn leads to a reorganization of the aqueous phase. Meanwhile, in the organic phase, the formation of a cavity is necessary to accommodate the metal, also requiring solvent reorganization to accommodate the metal and any co-extracted species. The metal itself may exhibit a preference for specific binding sites within the solvent, as well as possibly experiencing repositioning or reorientation. Increasing metal concentrations in the process adds another layer of complexity as the structuring of the organic phase can change, which may result in the metal being accommodated in different solvent sites, altering the solvent–metal interactions present, plus the possibility of clustering of the metal itself. Depending on the nature of the metal extractant, different extraction mechanisms can be expected and are summarised in Table 3. These are of course broad generalisation with the important influence of co-extracted solutes, the formation of larger-scale hydrogen bonded aggregates, and how these may influence the extraction mechanism not considered in the presentation of direct metal ion-ligand coordination in Table 3. For a more detailed discussion of extraction mechanisms, the reader is redirected to ref. 8, 68 and 86.

**Table 3:** Summary of extractant type, resulting predominant extraction mechanism, and hydrogen bonding influence on the metal complex and partition (L – ligand, M^*n*+^ – metal ion; X^−^ – salt counter anion; C^+^A^−^ – permanently ionic extractant)

Extractant type	General mechanism		Influence of H-bonding
Acidic extractant (HBD)	Cation exchange	• *n*HL_(org)_ + M^*n*+^_(aq)_ ↔ [ML_*n*_]_(org)_ + *n*H^+^_(aq)_	- Extractant deprotonation (pH swing)
			- Stabilisation *via* outer sphere H-bonding to form size selective cavities (*e.g.* phenolic oxime extractants)
			- Formation of stable hydrogen-bonded assemblies at higher extractant concentration (*e.g.* phosphoric acid extractants)
Basic extractant (HBA)	Anion exchange	• pH swing: *n*L_(org)_ + *n*H^+^_(aq)_ + MX_*y*_^*n*−^_(aq)_ ↔ [(LH)_*n*_MX_*y*_]_(org)_	- Extractant protonation (*e.g.* amines; pH swing)
		• Anion swing (for ionic DESs only): *n*[C^+^A^−^]_(org)_ + MX_*y*_^*n*−^_(aq)_ ↔ [(C)_*n*_MX_*y*_]_(org)_ + *n*A^−^_(aq)_	- Primarily driven by outer-sphere H-bonding interaction
			- Influenced by stability of metalate complex and Hofmeister series (charge density and energy of hydration)
Solvating extractant (HBA)	Ion pair	• *n*L_org_ + MX_*y*_ ⇌ [L_*n*_MX_*y*_]_org_	- Interaction through inner or outer sphere complexes (*e.g.* TBP extraction of uranyl ions, TODGA extraction of lanthanide nitrates)
			- Stabilisation of reverse micelle aggregates through H-bonding with polar solutes (water, counter anions)
			- Potential overlap with anion exchange depending on the extractant and aqueous phase acidity

Ignoring dehydration of the electrolyte (Δ*G*_hydration_), common to most hydrometallurgical approaches (*e.g.* adsorption, precipitation), Δ*G*_transfer_ can thus, in a simplified way, be decomposed into three main contributions (scheme in Fig. 6A). These are (i) the favourable complexation energy of the metal ion and extractant (Δ*G*_complexation_), which is partially quenched by (ii) solute confinement in the highly concentrated polar domains of the organic phase (Δ*G*_confinement_) and (iii) solvent-phase nanostructure reorganization around the extracted and co-extracted species (Δ*G*_structuration_).^68,89^ The solvent's structuring and the number of favourable interaction sites play a crucial role in determining both the efficiency and selectivity of extraction. To date, most research has focused on maximising the “hard” electrostatic interactions, namely the enthalpic contribution of Δ*G*_complexation_, through changes to the extractant chelating group. Comparatively, less attention has been paid to improving the SX selectivity by adjusting the weak interactions associated with the entropically driven structuration of the organic phase.

As structured solvent phases in SX are in dynamic equilibrium, considering only the first solvation sphere of the extracted ions provides a limited assessment of the SX free-energy landscape and does not fully exploit the potential stabilising forces over longer correlation lengths, such as second neighbour and percolation effects. Hydrogen bonding in the “soft” outer-sphere of “hard” metal–ligand complexes with non-ionic extractants plays a critical role in determining the selectivity of metal separation.^92,93^ Finally, H-bond donors are commonly added as phase modifiers to quench the critical phenomenon of third-phase formation in which the apolar phase splits into a heavy and light phase upon extraction of polar solutes above a certain concentration.^94–96^ The formation of a third-phase is deleterious to the design of cost-effective separations as it practically restricts metal loading of the organic phase below its theoretical saturation. Whether it is through the extraction of a polar solute with a hydrogen bonding capacity such as water or an acid,^97–99^ or through the deliberate addition of hydrotropic phase modifiers (*e.g.* propylene glycol monopropyl ether),^95,96^ the abundance of hydrogen bonds in the SX apolar phase is shown to swell the polar network volume, resulting in a gain of the solvent configurational entropy. In such cases, an increase in the extraction efficiency of various lanthanide cations was observed with no modification of the actual complexation energy, compensated for instead by (i) an increase in the dielectric constant of the organic phase due to the swelling of the polar domain and the consequent decrease of the charged solute Born energy, (ii) favourable intra-aggregate entropy of mixing, and (iii) the synergistic effect (in the case of hydrotrope–extractant mixtures). Such examples clearly highlight the potential of manipulating configurational entropy *via* the introduction of hydrogen bonds to improve extraction yields and solvent loading for more sustainable separation processes. Although more work is required to address the selectivity of this approach, harnessing the potential of soft interactions in SX may improve the difficult separations relying on small variations of complexation energies.

### 2.5. Potential advantages of DESs in SX

In conventional SX, a small concentration of extractant (∼0.1 to 0.5 mol L^−1^) is dissolved in an inert diluent often making up more than 90 mol% of the apolar phase. In such systems, the Δ*G*_structuration_ contribution is usually small and is dependent on the free energy of micellization of extractant monomers in the organic phase and the free energy of the chains. The latter represents the deviation of the actual packing parameter from the preferred one due to the presence of metal ions, water and/or acid in the polar core of the reverse micelle.^100^ Solvent restructuration in these “dilute” systems containing a large molar excess of diluent to extractant molecules is primarily attributed to metal-specific clustering upon the extraction of polar solutes.^101,102^ Comparatively fewer works address the possibility of tuning the selectivity of SX by controlling the dynamics and mesoscale organisation of an “extractant concentrated” organic phase, such as type V DESs.^103,104^ Control of the intermolecular interactions dictating the hierarchical self-assemblies in SX, and therefore the magnitude of the Δ*G*_structuration_ contribution, provides a new avenue for the design of new separation approaches.^103^

A proof of concept of the potential of non-ionic DESs for SX, illustrating the above discussion, is summarised in Fig. 6B and C. The non-ionic extractant TOPO may be incorporated as a DES component with either decanoic acid (C_10_OOH) or thymol and retain its metal-ligating properties.^47,63^ By a simple manipulation of phase structuration through the manipulation of the HBD selection and its molar fraction from *x*_HBD_ = 0.5 to 0.8, the distribution factor of palladium could be non-linearly altered (Fig. 6B). The decreased metal distribution with increasing *x*_HBD_ is assigned to antagonistic effects stemming from the change in the concentration of “free” non-hydrogen bonded TOPO available for metal extraction as determined by molecular dynamics. The latter depends on both *x*_HBD_ and the nature of the HBD (greater self-association of decanoic acid relative to thymol). Harnessing these non-linear extraction tendencies as a function of the HBD type, DES molar ratio and aqueous HCl concentration, a decorrelation in the separation factor between Pt and Pd was obtained in the TOPO + thymol system for *x*_TOPO_ = 0.4 and 2 mol L^−1^ HCl, as shown in Fig. 6C. After optimisation, this permitted a threefold increase in the selectivity of the Pt/Pd separation relative to the conventional SX system with the same extractant.^63,105^

The application of DESs in conjunction with their intrinsic properties may enhance the purity of the target elements while decreasing the environmental impact. Additional potential DES advantages include those listed below, some of which are discussed further in the next section.

(i) *Overcoming the solubility issue* of certain extractants in organic diluents. For example, TOPO + thymol or phenol have a wide liquidus range even at extractant concentrations of ≥50 mol%,^19,47^ in contrast to the limited TOPO solubility of 0.2 mol L^−1^ in kerosene.^106^

(ii) *Process intensification* due to the larger extractant concentration, enabling higher metal extraction and operating at lower organic to aqueous phase ratios.

(iii) *The absence of third-phase formation* arising from its intermolecular disorder (flexibility of molecular conformations). For example, while third-phase formation occurred for TOPO diluted in cyclohexane at Pt concentrations greater than 1.0 × 10^−4^ mol L^−1^,^105^ no such phase separation was observed in the DES even after the extraction of 0.1 mol L^−1^ Pt (a thousand-fold increase).^63^

(iv) *Elimination of petroleum-derived diluents* whilst anticipating the shift towards a more sustainable biorefinery context through judicious component selection, including cheap natural products (terpenoids, fatty acids, alcohols *etc*.).

(v) *No new compounds* are synthesised; instead, the process relies solely on the molar ratio of the components, thereby simplifying the economical, toxicological, and environmental assessment. By giving new life to otherwise poorly selective extractants, DESs circumvent the need for complex extractant design and synthesis, valorising instead extractant molecules already approved and characterised.

## 3. DES properties

### 3.1. DES structuration

A fundamental question for the application of any solvent is how its structure, which can vary over various length and time scales (*i.e.* local *vs.* bulk structure), controls the liquid property and potentially its performance? This is a non-trivial problem as the influence of the organic phase pre-organisation on traditional SX is often considered secondary to extractant reorganisation induced by the polar solute, as their introduction alters the balance of weak interactions that drive self-assembly in these dilute systems.^68^ An improved understanding of the causal relationship between DES structuration, the interactions that drive it, metal extraction and system selectivity is required to reach an *a priori* design of these solvents. It is important to highlight that the microstructure of any solvent is in dynamic equilibrium and that the properties measured are the average of multiple co-existing conformations. Reducing the analysis of DES structure to binary HBA–HBD combinations severely restricts any potential interpretation.

The inherent requirement for extracting ligands in SX to chelate metal ions whilst being poorly water miscible entails a degree of amphiphilicity, suggesting solvophobic effects as an important driver for self-assembly. Taking examples from well-studied simple amphiphiles like alcohols, the size, shape, and extent of percolation of the three-dimensional hydrogen-bonded network in type V DESs are likely dictated by a combination of the ratio of hydrogen bond donor and acceptor sites in the mixture, steric hindrance to packing, and minimisation of the free energy of curvature of metal-loaded extractant aggregates.^107^ Optimal networking occurs when the number of HBD sites matches the number of HBA sites, with any deviations from this ratio creating defects within the hydrogen bond network, resulting in a “looser” arrangement, which can enhance the DES fluidity. This was observed for example in the thymol + menthol DES due to the poor HBA nature of the phenolic oxygen, resulting in the presence of free non-hydrogen bonded thymol molecules in the mixture.^49^

**Fig. 7:**
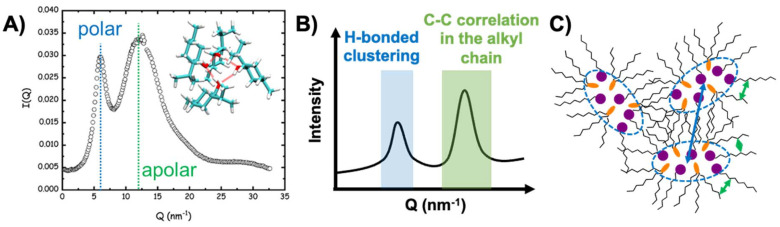
(A) X-ray scattering profile of the liquid thymol + menthol DES (*x*_thymol_ = 0.5, *T* ≈ 293 K); the inset shows a typical thymol–menthol cluster identified from MD simulations. This figure has been adapted from ref. 49 with permission from the American Chemical Society, copyright 2021. (B) Simplified schematic of the interaction assignment giving rise to the peaks in the scattering profile and (C) probable general structure of the mixed HBA–HBD cluster in type V hydrophobic DESs, with the colour code of arrows matching that of panel (B).

Due to the presence of only HBD and HBA species in DESs, the resulting hydrogen bonded network is highly connected, dense and complex. The liquid structure of non-ionic eutectic mixtures was probed by a range of spectroscopic techniques, often in combination, including infrared,^108^ Raman,^49,109^ and xenon NMR spectroscopy,^110^ molecular dynamics (MD) simulations,^49,108,111–113^ dielectric spectroscopy,^114^ and small angle X-ray or neutron scattering (SAXS/SANS),^49,108,109,115^ including at high pressures.^116^ All studies indicate the existence of dynamic heterogeneities and mesoscale ordering, with the emergence of polar and apolar segregated domains. SAXS analysis of the thymol + menthol and TOPO + decanoic acid systems^49,109^ showed the presence of a pre-peak (Fig. 7A) appearing before the standard peak associated with neighbouring alkyl moieties, as schematically generalised in Fig. 7B. Such a pre-peak feature is usually assigned to intermediate-range ordering as the correlation length is greater than the first shell of neighbouring molecules. Using the liquid structure of monohydroxy alcohols with long alkyl chains such as octanol as an example,^117^ the pre-peak was assigned to the emergence of an intermolecular polar domain and the formation of hydrogen bonded chain-like prolate clusters.^109^ Contrary to liquids with reduced packing constraints like methanol or mixtures of short-chain molecules (glycerol + lactic acid),^115^ steric exclusion between the larger HBA and HBD precursors in hydrophobic eutectic mixtures inhibits the formation of a continuous polar domain, yielding instead smaller hydrogen bonded clusters with a hydroxyl rich core and an alkyl rich exterior, as represented in Fig. 7C. This is coherent with the conclusions from SANS analysis of the menthol + decanoic acid mixture, which reveals the presence of micellar-like spherical aggregates.^115^ Importantly, classical MD simulations using non-polarizable forces could adequately reproduce the X-ray scattering profile of non-ionic eutectic mixtures, emphasising its potential for studying the structuration of these solvents.

As evidenced by the presence of polar/apolar segregation in pure solvents (*e.g.* fatty alcohols) or ideal mixtures (*e.g.* menthol + decanoic acid or butylated hydroxytoluene (BHT) + menthol), the emergence of mesoscale ordering is not a consequence of solvent non-ideality but rather occurs in certain molecular “fragile” glass-forming liquids.^114^ Questions remain as to if and how the structure of true non-ideal DESs differs from that of simple eutectic mixtures.^129^ Xe-NMR chemical shifts and longitudinal relaxation times suggest a decrease in the free volume of DESs relative to ideal mixtures (tighter packing of the components).^110^ However, due to the different structures of the HBAs and HBDs tested, it is unclear if this effect is solely due to non-ideality or simply a change in the structure of the precursors. In an excellent contribution, D'Hondt and Morineau studied the molecular dynamics of the thymol + menthol system by dielectric spectroscopy and DSC.^114^ They showed that upon approaching the glass transition temperature, the glassy dynamics of the DES were slower than anticipated for ideal mixtures due to spatial heterogeneities, an observation further confirmed by the dipolar relaxation dynamics of the solvent. Furthermore, the dielectric strength of the solvent varied with composition with a maximum for approximately *x*_thymol_ = 0.5, suggesting that the equilibrium between different populations of hydrogen bonded clusters shifted from cyclic to a more linear structure due to thymol–menthol interactions.

A final question remains how the structure of type V DESs varies under practical SX applications as most studies to date have been performed on pure mixtures. Important aspects include how polar and ionic solutes are distributed in the solvent upon extraction and if they lead to any changes in preferential interactions and liquid structuring. Although preliminary, SAXS spectra of the TOPO + decanoic acid saturated phase after contact with a HNO_3_ solution of lanthanide cations show a shift in the pre-peak position but no change in the low *Q* region, indicative of a swelling of the polar domain and homogeneous dispersion of the co-extracted solutes in the DES phase. Furthermore, the SAXS spectra presented a new intermediate peak at *Q* values between that attributed to hydrogen bond clustering and apolar adjacency, assigned to ion–ion correlations arising from extracted lanthanides.^109^ Under such conditions, Raman analysis found an increase in both the population of metal complex bound TOPO and the formation of carboxylic acid dimers; the extent of this reorganisation was dependent on the starting molar composition of the DES. Evidence suggests the partial disruption of the preferential interaction in the pure DES under SX conditions in favour of TOPO adducts with HNO_3_ and lanthanide complexes at the expense of decanoic acid.^109^

### 3.2. Hydrodynamic properties

Hydrodynamic properties, namely viscosity, density and interfacial tension, are key to efficient emulsification and demulsification in industrial mixer–settler stages, which often rely solely on gravity. These parameters influence the coalescence rate of the phases and ultimately determine the settling time, representing the time required for complete phase separation.^118,119^ This in turn can impact entrainment losses and negatively impact the SX process economically and environmentally. As a general rule of thumb, the rate of phase separation can be increased by minimising the difference in viscosity (*η*) by reducing the organic phase viscosity, increasing the difference in density (*ρ*), or increasing the interfacial tension (*σ*) between organic and aqueous phases.

**Fig. 8:**
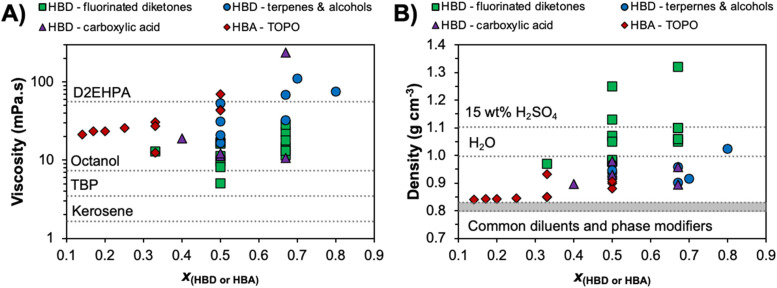
(A) Viscosity and (B) density of hydrophobic eutectic mixtures at 298 K as a function of composition and type, with dashed lines indicating the respective property of commonly used solvents in SX. Viscosity and density data are available in Table S4.†

The viscosities of various non-ionic eutectic mixtures at 298 K as a function of their composition and type, divided into DESs containing fluorinated diketones, carboxylic acid, terpenes, and TOPO, are presented in Fig. 8A and listed in Table S4.† Some overlap between categories is unavoidable and in such cases the ligand dictating metal extraction determines the categorisation (TOPO + thymol DES is categorised as HBA – TOPO in Fig. 8, and not under HBD – terpene). It is important to note that the presence of water, a variable that is not always controlled for and/or reported, can greatly reduce the viscosity of the DES relative to the anhydrous solvent and skew tendencies. For comparative purposes, the viscosity of common diluents and extractants in SX are also included, including kerosene (1.64 mPa s), tributylphosphate (TBP, 3.46 mPa s), 1-octanol (7.36 mPa s), and di(2-ethylhexyl)phosphoric acid (D2EHPA, 56 mPa s). Generally, viscosities of type V DESs are significantly lower than their ionic DES counterparts at room temperature (in the range of 265 to 783 mPa s for quaternary ammonium halide salts with decanoic acid),^14^ but greater than the equivalent diluent phase in conventional diluent SX. Reported eutectic viscosities span one order of magnitude, ranging from 5.04 mPa s for the mixture of HTTA + TBP^120^ to 110.4 mPa s for menthol + borneol,^121^ with most falling in the range of 15 to 60 mPa s. Greater than expected viscosities can result from systems presenting partial proton transfer, as in the case of lidocaine + decanoic acid (237.5 mPa s)^43^ or for temperature/compositions close to the SLE due to the exponential increase in viscosity. In turn, the introduction of fluorinated functional groups in the DES constituents, the addition of water,^48,121^ or the increase of temperature^122^ can all significantly lower viscosities to values in line with some SX phase modifiers.

Additional contributions to viscosity arise from the molecular structure of the components due to its influence on the packing parameter, and the deviations from ideality of the eutectic phase. Alhadid *et al.*^122^ showed that the viscosity of typical pure constituents followed a general tendency based on their molecular structure, namely cyclohexyl > phenyl > linear. In most cases, the viscosity of the mixtures is in between (or higher than) the viscosities of the pure components, with excess viscosities typically observed for non-ideal systems. For example, the viscosities of pure menthol and thymol at 328 K (the temperature at which both are liquid) are 7.19 and 3.6 mPa s respectively.^46,121^ At this temperature, the predicted viscosity of the type V DES menthol + thymol (*x*_menthol_ = 0.5) based on the ideal mixture rule (ln(*η*_mix_) = *x*_1_ ln(*η*_1_) + *x*_2_ln(*η*_2_)) is 5.3 mPa s, 18% lower than the value of 6.43 mPa s experimentally measured.^121^

The density of non-ionic eutectic mixtures at 298 K according to the same classification previously described is presented in Fig. 8B and compared against the typical density range of organic diluents (kerosene, paraffin, octanol), water, and acidified water with 15 wt% H_2_SO_4_. A wider variability is observed for reported type V DES densities relative to their hydrophobic ionic counterparts, with most quaternary ammonium halide-based mixtures presenting densities in the range of 0.89 to 0.94 g cm^−3^.^14,123^ TOPO-based mixtures appear to be promising for SX based on density values, presenting on average the lowest values in the range of some phase modifiers and diluents. Alternatively, mixtures containing fluorinated constituents possess the greatest density, in some cases exhibiting large differences from the density of the aqueous phase although a phase inversion is observed relative to common SX operation (*ρ*_DES_ > *ρ*_H_2_O_). The application of terpene- or carboxylic acid-based mixtures could pose some issues due to their similar densities to that of non-acidified water. As in any mixture, the final properties are dictated by the choice of precursor. For example, aromatic diluents typically have higher densities than aliphatic diluents, so careful selection of the DES component can impede or promote dispersion and coalescence.

A notable absence from the discussion so far is the interfacial tension of DES–water systems, which to the best of our knowledge has not yet been reported. The air–DES surface tension was previously measured for a limited number of systems, including menthol or thymol with decanoic acid^124,125^ and terpene–terpene systems.^112^ The surface tension in such a system is in the range of 22.4 to 31.3 mN m^−1^, comparable to that of 1-decanol (28.0 mN m^−1^). Nevertheless, this provides limited information on the interfacial tension and the DES–water interfacial structure, which are crucial to understand the selectivity in practical SX applications often operating under non-equilibrium conditions.^126^ This is particularly complex in type V DESs based on components with large differences in aqueous solubilities such as TOPO + malonic acid,^20^ as it can influence the preferential interfacial accumulation of one constituent over another, promoting a significant difference in the interfacial *vs.* bulk structure. Furthermore, according to capillary wave theory, density differences and interfacial tension between two coexisting phases influence interfacial thickness (*ζ*, eqn (8)),^127^ potentially yielding a significant expansion of *ζ* relative to that in conventional SX that is in on the order of a couple of nanometers.^128^8
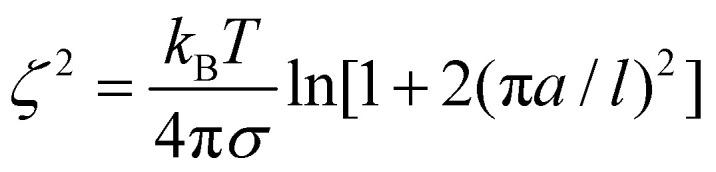
where *k*_B_ is the Boltzmann constant, *a* is the capillary length, and *l* denotes the average diameter of water molecules. Although no data are available for interfacial tension, expected values can be estimated from reported data. Due to the amphiphilic nature of most reported DES components and their structural similarity to common organic solvents, DES/H_2_O interfacial tension is expected to be in the range of 4 to 35 mN m^−1^ depending on the hydrophilicity of the chosen components (1-butanol – 1.8 mN m^−1^; 1-octanol – 8.52 mN m^−1^; cyclohexanol – 3.92 mN m^−1^; benzyl alcohol – 4.75 mN m^−1^; octanoic acid – 8.5 mN m^−1^; ethyl hexanoate – 19.80 mN m^−1^; 2-octanone –14.09 mN m^−1^; TBP – 28.5 mN m^−1^).^129,130^

**Fig. 9:**
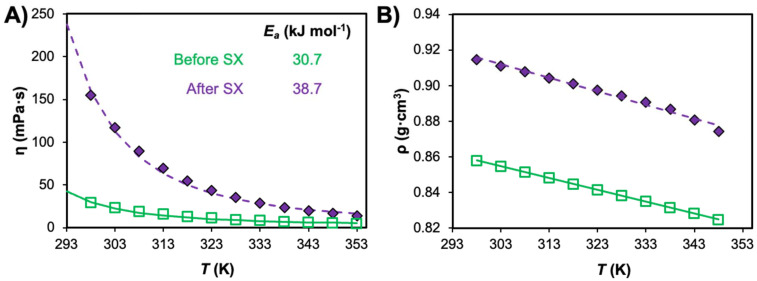
Evolution in the (A) viscosity and (B) density of the TOPO + decanoic acid system (*x*_TOPO_ = 0.5) as prepared (before SX) and after metal ion loading (Co^2+^ and Mn^2+^ mixture) of 44 g L^−1^ in the DES phase; data are taken from refs. 47 and 131. The activation energy of viscous flow (*E*_a_) was also calculated from the viscosity data and provided in the inset.

A final consideration is the influence of co-extracted solutes after SX application on the final properties of the solvent. As detailed in the previous section, ion–ion correlations can emerge in the DES phase at saturation, significantly disrupting the initial DES structure. Such structural changes are reflected in the hydrodynamic properties of the metal-saturated DES, as shown in Fig. 9 for TOPO + decanoic acid after saturation (Co^2+^ and Mn^2+^ ion mixture).^131^ A threefold increase in viscosity is observed relative to the as-prepared system as well as a non-negligible increase in density of Δ*ρ* = 0.06 g cm^−3^. This highlights the difficulty in extrapolating pure DES properties to realistic SX conditions and suggests that, despite inherent liquification properties of DESs and greater possible extractant concentrations, a compromise between solvent loading and hydrodynamic properties is required. Finally, general tendencies of the aqueous phase composition on the interfacial surface tension can be derived from conventional extractants in diluent systems. Interfacial tension typically increases with aqueous salt concentration, the extent of which depends on the salt properties (salting-in *vs.* salting-out) according to the Hofmeister series. Unfortunately, the influence of acid concentration is more difficult to ascertain as this is shown to vary non-linearly with acid concentration and the extractant and phase modifier combination, although lower interfacial surface tensions are generally observed in strongly acidic solutions.^130,132,133^

### 3.3. DES stability and environmental impact

The investigation of eutectic solvents is frequently justified based on their perception as “green” solvents with high biodegradability and low toxicity due to the potentially natural origin of their precursors – giving rise to the label NADES, often neglecting that “natural” compounds such as menthol are primarily produced synthetically.^134^ Furthermore, conceptual legacies from the original introduction of DESs as substitutes for ionic liquids persist, namely that these are economical and stable solvents with low volatility. The molecular nature of type V DESs being distinct from that of ionic liquids and type I to IV DESs suggests that such assumptions must be revised.

Despite their extensive academic investigation, no systematic study could be found that evaluated the long-term chemical stability of type V DESs. Rather, most work confirms their stability *via* FTIR or NMR spectroscopy directly after preparation and occasionally after a limited cycle of extraction and regeneration.^135^ Organic phases subjected to realistic continuous SX conditions are confronted with aggressive environments of high salinity, high acid concentrations, the presence of oxidizing or reducing agents, and/or ionizing radiation. Such conditions give rise to several potential degradation pathways *via* oxidation, condensation, acid hydrolysis, radiolysis, photodegradation, Beckmann rearrangement, and nitration to name but a few.^62,136–138^ The accumulation of degradation products contributes to changes in the extraction efficiency and selectivity as well as entrainment losses. Unfortunately, anti-degradant sacrificial agents sometimes added to SX formulations to protect the extractant from hydrolysis, oxidation, or nitrification are typically based on alkylphenols and alcohols^139^ and share similarities with common constituents of type V DESs. Whilst the presence of hydrogen bonds protecting functional groups under attack can slow the kinetics of degradation, this is not expected to hinder it. Due to their reactivity and anti-oxidizing properties, phenolic components such as thymol must be monitored carefully if extended use is anticipated. Finally, esterification reactions between DES mixtures composed of alcohol and carboxylic acid components are expected (*e.g.* menthol + carboxylic acid mixtures),^140,141^ with the conversion yield being influenced by the presence of catalysts (including inorganic acids), increased temperature, and an excess of acid.

**Fig. 10:**
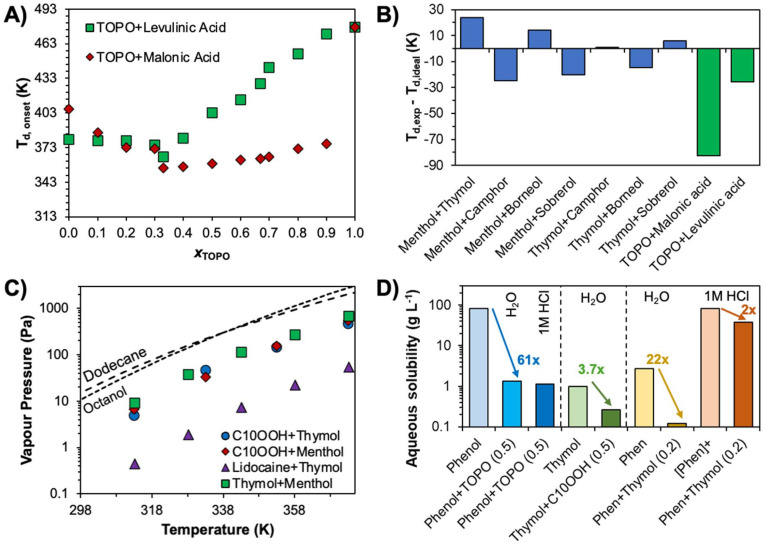
(A) Onset of thermal decomposition (*T*_d_) for TOPO + malonic acid and TOPO + levulinic acid mixtures across the whole compositional range determined by dynamic thermogravimetric analysis. Adapted from ref. 20 with permission from the Royal Society of Chemistry, copyright 2020. (B) Difference in the experimentally measured *T*_d,exp_ and theoretical *T*_d,ideal_ from the ideal mixing rule for different hydrophobic acid mixtures with equimolar composition.^20,121^ (C) Total vapor pressures of hydrophobic eutectic mixtures with equimolar composition compared against organic diluents.^142,143^ (D) Comparison of the aqueous solubilities at 298 K of pure constituents against those incorporated into eutectic mixtures for phenol,^19^ thymol,^46^ and 1,10-phenanthroline^48^ as a function of the aqueous phase acidity.

More information is available regarding the thermal stability of type V DESs, determined by thermogravimetric analysis (TGA); the influence of composition and system selection is illustrated in Fig. 10A and B respectively. Byrne and co-workers presented a detailed study on the impact of composition on the thermal stability of TOPO + malonic acid and TOPO + levulinic acid mixtures, with Fig. 10A showing a distinct non-linear evolution of the onset degradation temperature (*T*_d_) between the values of the pure components.^20^ A two-step decomposition process was observed by dynamic TGA in both systems for all intermediate compositions, with the *T*_d_ value being determined by the least stable component of the mixture (the carboxylic acid). Furthermore, antagonistic effects of the mixture on its stability are apparent as some compositions present experimental *T*_d_ values far below those predicted by the ideal mixing rule from the value of the pure components. A comparison of these values with those reported for terpene + terpene mixtures^121^ in Fig. 10B presents a more nuanced picture. Generally, *T*_d_ values remain lower than or between those of their pure constituents, although with smaller deviations than for carboxylic acid systems. In contrast, thymol mixtures exhibiting strong non-ideality presented improved stability, with experimental *T*_d_ values above those of the pure individual HBA and HBD. Whilst this suggests the formation of azeotropes, no such behaviour was reported in the vapor–liquid equilibrium of the thymol + menthol mixture,^49^ and thus requires further investigation. Importantly, the *T*_d_ values discussed are based on dynamic TGA experiments of as-prepared DESs in the absence of co-extracted solutes under an ambient atmosphere, which can overestimate the stability. Overall, the DES thermal stability is dictated by the least stable component, although to what extent appears to be system and composition dependent.

The volatility of selected non-ionic DESs is compared against common organic solvents in SX in Fig. 10C.^142,143^ Predictably all systems are volatile, with the total vapor pressure in DESs essentially being determined by the partial vapour pressure of the most volatile component. In the terpene + carboxylic acid and terpene + lidocaine systems, the terpene constitutes ≥90% of the total vapour pressure of the system, with menthol being more volatile than thymol. Encouragingly, it appears that some DESs present much lower vapor pressures than common diluents such as 1-octanol, *n*-dodecane, or toluene (but far greater than ionic liquids). However, a limited number of longer-term stability studies were performed and showed non-negligible to considerable mass losses with time. For example, isothermal TGA of the same mixtures as those in Fig. 10A for *x*_TOPO_ = 0.33 at 363 K for 22 h resulted in mass losses of 37% and 20% for malonic and levulinic acid DESs, respectively, with the former corresponding to the complete loss of malonic acid from the system.^20^ Unfortunately, the tendency of many organic acids to sublime in addition to decomposing^144^ suggests that some reported type V DESs are ill-suited to higher temperature operations. This can be mitigated by changing the nature of the HBD, as the TOPO + decanoic acid mixture (*x*_TOPO_ = 0.5) presents a weight loss of only 3.4% after 6 h at 377 K.^131^

Beyond gaseous emissions, an important pathway impacting DES bioavailability and contamination is through losses to the aqueous phase upon mixing. The aqueous solubilities of various pure compounds are compared to those obtained upon their inclusion in DESs; the results are shown in Fig. 10D. Regardless of the system, enhanced hydrophobicity is observed, exceeding one order of magnitude for phenol after the addition of TOPO.^19^ Greater relative reductions in water partition are observed for components presenting higher initial water solubilities as well as for systems with negative deviations from ideality (TOPO + phenol^19^ and to a lesser extent 1,10-phenanthroline + thymol^48^). Final aqueous solubilities are comparable to or below that of 1-octanol (0.2 g L^−1^ at 293 K) but larger than those of alkane diluents (0.01 g L^−1^ for decane at 293 K).^145^ Nevertheless, these compare favourably with ionic DESs, where the solubility of the salt component in the aqueous phase after contact varies from 15.5 wt% for tetrabutylammonium chloride to 1.1 wt% for tetraoctylammonium chloride when combined with decanoic acid.^14^ Importantly, final solubilities are greatly dependent on the aqueous phase pH. The solubility of protic compounds is expected to increase for pH nearing or greater than their p*K*_a_, whilst the contrary holds for components with protonable moieties, as demonstrated in Fig. 10D for 1,10-phenanthroline upon contact with 1 mol L^−1^ HCl. Careful pH control is required based on the DES selection to minimise losses and emulsification.

Despite DESs being labelled as green solvents, there is surprisingly little information to back up the claim. The limited data on the topic underscore the need to avoid generalities when discussing these solvents and to not consider DESs as singular entities. As for any mixtures, the resulting properties are subject to those of the pure components and can present antagonistic or synergistic phenomena depending on the property in question, the system selection, and the molar composition. Under ambient temperature conditions, DESs appear to be promising alternatives to organic phases in conventional SX if carefully selected, although significant work is required to understand their long-term chemical stability, biodegradability, and toxicity. This is of particular concern for the growing use of DESs incorporating fluorinated β-diketone constituents. Comparative life-cycle assessment studies are essential to categorically ascertain the environmental merit of type V DESs.

### 3.4. Solvatochromic parameters – DESs as diluents in SX?

The discussion has focused on binary eutectic mixtures in which the extractant is also the phase former. However, some recent works on the extraction of lithium,^146^ strontium,^147^ indium,^148^ thallium,^148^ and REEs^149–151^ investigated the use of ternary mixtures to improve the extraction characteristics (extraction yield, usable pH range, or viscosity) or selectivity, often through the addition of an alcohol component. An incomplete description of extraction tendencies in the different permutations of binary mixtures can make it difficult to ascertain the contribution of each component in the ternary mixture to extraction, particularly as the extraction mechanism is often assigned to one or two system components. Furthermore, the addition of a tertiary component as a co-solvent can enhance or weaken existing hydrogen bond interactions,^152^ such that it is challenging to determine if any “synergistic” effects are due to mixed ligand ion complexes or simply the greater availability of the original extractant. However, this approach raises an interesting question, namely can a non-coordinating DES substitute common diluents in SX and what diluent(s) could it replace?

Whilst the diluent is often considered as an inert medium that does not actively contribute to ion extraction, this was shown to be a simplification with distribution ratios varying based on the solvent characteristic.^153^ A number of solvent descriptors are available to reflect variations in polarity, hydrogen bonding capacity, dielectric constant, and “solvating properties” of different diluents. The most popular solubility parameters include the Hildebrandt parameter, Hansen parameters, Kamlet–Taft parameters, and Abraham parameters; with more details on the various parameters available elsewhere.^154^ Although certain structure–property relationships between descriptors and thermodynamic parameters and/or extracted complex stoichiometry were observed, the nature of the correlation is usually elusive and specific to a given system. Nevertheless, certain general trends are typically accepted. The energy of cavity formation, provided by the Hildebrandt parameter, as well as polarity of the diluent were shown to influence the extractant–diluent interaction and the degree of extractant self-association and polymerisation (monomer, dimers, higher order oligomers).^155,156^ As such, aprotic low-polarity solvents are often favoured as diluents, although some exceptions exist. It is worth noting that several regression tools for the prediction of organic solvent parameters, including DESs, are available in the literature for the estimation of new mixtures.^157^

**Fig. 11:**
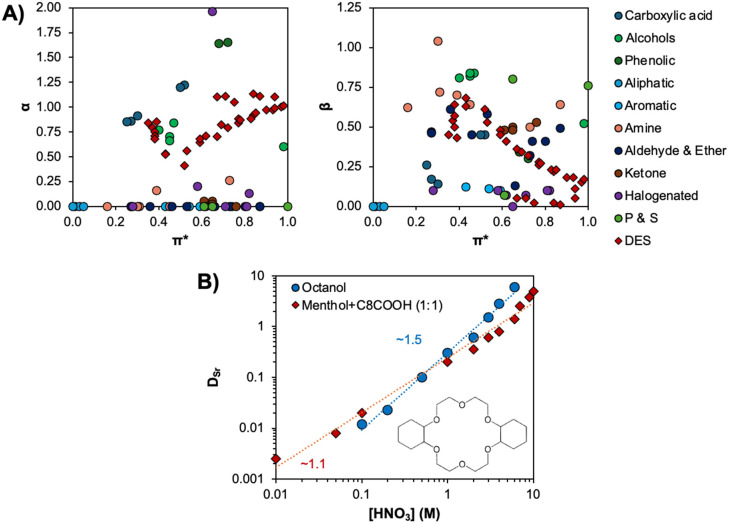
(A) Plot of binary experimental Kamlet–Taft parameters of organic solvents and non-ionic eutectic solvents. Organic solvent parameters were obtained from the Marcus database,^154^ whilst those of DESs are available in Table S5.† (B) Comparison of the effect of nitric acid on the solvent extraction of Sr^2+^ by 0.1 mol L^−1^ dicyclohexano-18-crown-6 ether in either octanol or a mixture of nonanoic acid and menthol. This figure has been adapted from ref. 147 with permission from Elsevier, copyright 2022.

A popular solvent scale is given by the Kamlet–Taft parameters, *α*, *β*, and π*, which provides information regarding the hydrogen bond donor (*α*) and acceptor (*β*) capacity of the solvent, as well as its polarizability/dipolarity (π*). The Kamlet–Taft parameters reported for hydrophobic eutectic mixtures are given in Fig. 11A and superimposed over the parameters of common organic solvents taken from the Marcus solvent database.^154^ As expected from the dominant nature of hydrogen bond interactions in eutectic mixtures, these solvents can be considered as protic solvents, as generally *α* > 0.5,^158^ but present a greater degree of variability in their hydrogen bond basicity. Furthermore, all eutectic solvents present moderate to high polarizability, with π* values from 0.35 to 0.98. Overall, it can be observed that non-ionic eutectic solvents occupy approximately the same chemical space as alcohols and carboxylic acids for *α*, and extend the range of these solvents for *β* to that of amines and ethers. This is likely, in part, due to bias in the data as Kamlet–Taft parameters are only available for carboxylic acid–terpenes and terpene–terpene mixtures, providing a limited representation of potential HBA–HBD combinations. Of interest, Martins *et al.* showed that for a given eutectic mixture the solvent characteristics could be further adjusted by altering the composition, with the Kamlet–Taft parameters varying approximately linearly between the values of the pure components in the thymol + menthol and thymol + camphor systems.^121^

In detailed contributions, the use of menthol + alkylcarboxylic acid mixtures was compared against kerosene as diluents for the extraction of In^3+^ and Tl^+/3+^ using D2HEPA as an extractant,^148^ and against fatty alcohols and hydrophobic ionic liquids for the extraction of alkali and alkaline Earth cations using dicyclohexano-18-crown-6 ether.^147^ Extraction tendencies using a crown-ether in the eutectic mixtures were similar to those obtained in alcohols, as shown in Fig. 11B, albeit generally exhibiting reduced distribution factors but comparable or greater Sr/Na and Sr/Ca selectivities. The use of ionic liquids as diluents provided a distinct extraction behaviour relative to molecular diluents and type V systems, further reinforcing that the latter should be viewed as an extension of organic solvents. Extraction in the menthol + decanoic acid system with the addition of D2HEPA mirrored tendencies in the equivalent system with kerosene.^148^ Smaller distribution factors were obtained for In^3+^ at lower aqueous phase acidities when extraction *via* cation exchange dominated but provided superior extraction for neutral species at higher acidity (solvation reaction mechanism), most likely due to the increased polarity of the eutectic solvent. Interestingly, the eutectic diluent system provided greater distribution values for Tl^3+^ over the entire acidity range. These results suggest the potential of DESs as diluents under specific conditions. However, the absence of significant changes to the extraction tendencies with common protic and aprotic solvents belies their “tuneable” characteristics. It is therefore debatable if the increased system complexity upon substitution of a conventional molecular solvent by a DES warrants their general application as diluents in SX, granted this does not exclude their application for specific SX systems. More complete studies incorporating experimental data coupled with LCA and techno-economic analysis are required to determine the viability of DESs as diluents.

## 4. Future perspectives

**Fig. 12:**
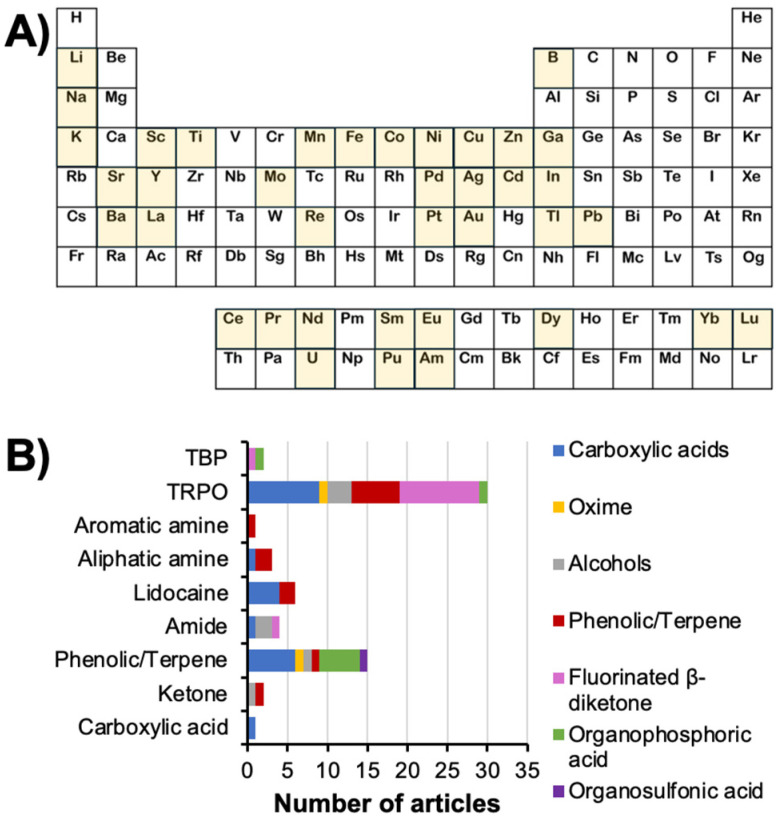
(A) The metal ions studied for type V DES extraction are highlighted, and (B) a breakdown of the DES system applied as a function of the HBD and HBA type (TRPO – trialkylphosphine oxide). Articles were obtained from Web of Science (06/12/2024) from 2019 onwards using the keywords “eutectic solvents” “metal” “solvent extraction” and further refined with “hydrophobic” and “non-ionic”.

Since the recent introduction of type V DESs in 2019, the extraction of over 35 metal ions was studied, as illustrated in Fig. 12A and Table 4, with Li^+^, Cu^2+^, REE^3+^, Fe^3+^ and Ni^2+^ being the most frequently investigated and to a lesser extent PdCl_4_^2−^, UO_2_^2+^, and Ti^3+^. Some of these could significantly benefit from the intrinsic nature of these solvents due to similarities with current SX processes. Notable examples include the SX of boron, which relies on alcohol extractants,^159^ the extraction of uranyl cations by phosphine oxide or phosphate extractants,^19^ or that of lithium, which often requires the use of synergistic mixtures of acidic and neutral extractants.^21,120^ Such rapid expansion attests to the vitality and growth of the research area. The inherent properties of type V DESs (liquification and overcoming extractant solubility issues) when applied to SX are yielding encouraging results regarding the avoidance of third-phase formation, rapid extraction kinetics, process intensification, and equal or greater separation factors than those of conventional SX. Furthermore, selected works demonstrated that the regeneration of the DES phase could be achieved by stripping (back-extraction) of extracted ions from the loaded DESs to an aqueous one. In general, approaches from conventional SX for a given extractant–metal ion separation can be applied to an equivalent DES system. It includes pH changes for extraction that occur through a change in the extractant's protonation state, or through altering metal speciation by varying the aqueous phase composition and/or introducing competing chelating agents for ion-pair extractions (*e.g.* EDTA). However, the greater extractant concentrations in eutectic solvents relative to conventional SX can require more aggressive stripping conditions, particularly for highly stable metal ion–extractant complexes, as observed in the incomplete stripping of transition metals from the thymol + 1,10-phenanthroline DES.^48^ Under continuous operation this can quickly lead to saturation of the eutectic phase and reduced extraction efficiency.

**Table 4:** List of type V DES systems applied as a function of the HBD and HBA type. Some systems are grouped together due to their proximity and are indicated with a slash (*e.g.* phenol/thymol)

HBD	HBA	Ions studied	Ref.
*N*-Alkyl alcohol	TOPO	[PtCl_6_]^2−^, Co^2+^, Cu^2+^, Fe^3+^, Dy^3+^, Nd^3+^, Pr^3+^, Sm^3+^	150, 160 and 161
Phenol/thymol/*tert*-butylphenol/menthol	TOPO	Dy^3+^, Nd^3+^, Sm^3+^, Co^2+^ UO_2_^2+^	19 and 162–164
*N*-Alkyl carboxylic acid	TOPO	Li^+^, La^3+^, Ce^3+^, Pr^3+^, Nd^3+^, Eu^3+^, Gd^3+^, Y^3+^, Lu^3+^, Fe^3+^, Co^2+^, Ni^2+^, Mn^2+^, Cu^2+^, [PdCl_4_]^2−^, [PtCl_6_]^2−^, Mo, Re	47, 109, 131 and 165–167
HTTA/HBTA	TOPO/TBPO	Li^+^, UO_2_^2+^, Am^3+^, Pu^4+^, Nd^3+^, Eu^3+^, Dy^3+^, Sm^3+^, Al^3+^, Fe^3+^, Co^2+^, Ni^2+^, Mn^2+^, Cu^2+^	21, 120, 146 and 168–172
Oxalic acid/malonic acid/levulinic acid	TOPO	Ga^3+^, Am^3+^, Eu^3+^, UO_2_^2+^	20 and 173
Bis(2,4,4-trimethylpentyl) dithiophosphinic acid	TOPO	Pr^3+^, Nd^3+^, Eu^3+^	149
Salicylic acid/3,5-dinitrosalicylic acid	TOPO	Yb^3+^, Lu^3+^	174
*N*-Lauroylsarcosine	TOPO	Sc^3+^, Y^3+^, Fe^3+^	175
Decanoic acid	Octanoic acid	Cu^2+^, Co^2+^, Ni^2+^	176
*N*-Alkyl alcohol	*N*,*N*-Dialkylacetamide	Ti^3+^	177 and 178
Decanoic acid	*N*,*N*-Di-butylacetamide	[AuCl_4_]^−^	179
Decanoic acid	Lidocaine	Co^2+^, Ni^2+^, Mn^2+^, Zn^2+^, Cu^2+^, Fe^3+^, Li^+^, Na^+^, K^+^, [AuCl_4_]^−^, [PdCl_4_]^2−^	180–184
*N*-Alkyl carboxylic acid	Menthol/thymol	Li^+^, Na^+^, Sr^2+^, Ba^2+^, Cd^2+^, Mg^2+^, Mn^2+^, Cu^2+^, Pb^2+^, Co^2+^, Ni^2+^, Mn^2+^, Zn^2+^, Fe^3+^	24, 147 and 185–187
Mandelic acid	Menthol	Cd^2+^, Cu^2+^, Pb^2+^	188
Salicylaldoxime/salicylic acid	Menthol	Cu^2+^, Ag^+^	189 and 190
Camphor-10-sulfonic acid	Menthol	Pb, Cd, Hg, As	191
2-Ethylhexylphosphonic acid mono-(2-ethylhexyl) ester	Menthol	Co^2+^, Mn^2+^, Cu^2+^	192
2-Methyl-2,4-pentanediol	Thymol	B (as H_3_BO_3_)	159
Menthol/thymol	Lidocaine/proton sponge	In^3+^, [AuCl_4_]^−^, [PdCl_4_]^2−^, [PtCl_6_]^2−^	23 and 193
Menthol	Camphor	[AuCl_4_]^−^	194
Menthol	*n*-Alkyl ethylenediaminium	Co^2+^, Ni^2+^, Cu^2+^	195
Thymol	H_2_O	Co^2+^, Ni^2+^, Cu^2+^	196
Thymol	1,10-Phenanthroline	Co^2+^, Ni^2+^, Mn^2+^, Zn^2+^, Cu^2+^, Fe^3+^, Cd^3+^, La^3+^, [PdCl_4_]^2−^	48
D2EHPA	Menthol	Co^2+^, Mn^2+^, Cu^2+^, Ni^2+^, Al^3+^, In^3+^, Tl^3+^	148, 197 and 198
D2EHPA	TBP	Li^+^, Al^3+^, Cu^2+^, Fe^3+^	199
Decanoic acid/decanol	Bis(2-ethylhexyl) amine	Ln^3+^	151
*N*-Alkyl alcohol	Camphor/hydroxypropiophenone	Fe^3+^	200

However, to date SX experiments have been performed using a rather limited number of type V DESs considering the possible range of HBD–HBA permutations available, providing a partial view of the chemical separation space and the potential of this emerging class of solvent. Due to their relative simplicity (monodentate ligand), stability, and excellent HBA capability, trialkylphosphine oxide extractants (TRPO) serve as good model compounds to study SX in type V DESs, hence their dominant utilisation, as summarised in Fig. 12B. However, much like Cyanex 923 is not applied to all SX systems, it is improbable that the inclusion of TRPO ligands in DESs is the panacea for complex SX separations. Despite care to source references as widely as possible in the various discussions of type V DESs presented, the bias arising from the dominant application of a small number of systems suggests that future updates are advisable as more results become available.

Hydrophobic DESs are not a universal solution to all SX challenges, as the choice of system depends on numerous factors such as the nature of the solute and contaminants, their speciation, and the composition of the aqueous and organic phases. However, when effectively utilized, they have the potential to offer an additional approach for addressing both current and future SX issues. Moving beyond trial-and-error applications of a few systems, there is the need for a fundamental analysis of system characteristics and their impact on SX. The starting point, as for all DES related studies, is the accurate description of the SLE phase diagram and when possible, the obtention of excess properties. For SX, systematic comparison with conventional systems for the same extractant is essential to appreciate if, and how, extraction mechanisms and properties are altered. This is particularly relevant as, although the effects of hydrogen bonding on the structure of DESs are mentioned, it is not yet clear how these interactions affect the partition coefficient and separation selectivity of metal ions. With this knowledge, several open questions can start to be addressed:

- *Is solvent non-ideality relevant for an application?* If the objective is to maximize extraction percentage, as is common in decontamination or analytical applications, rather than the separation selectivity, a truly non-ideal DES could be counterproductive.

- *Is there a link between DES non-ideality and antagonistic extraction tendencies?* How does the use of extractants as both the chelating agent and DES phase former impact the complex stoichiometry and atomic efficiency of ion-partition?

- *Is the DES non-ideality reflected in the liquid phase nanostructuration and properties?* Measurement of excess properties is recommended as well as more works on the evaluation of dynamic ones. A natural extension of this question is to what extent the structure is altered under SX conditions, particularly inter-molecular DES hydrogen bonding relative to competing binary extractant–ion or extractant–acid interactions. Is the structuration preserved upon dilution and how does it influence extraction?

- *What are the impacts of secondary contributions beyond hydrogen bonding?* The structure of DES components is often neglected at the expense of hydrogen bonding. However, DESs are sterically crowded solvents, with the number of functional groups and denticity, the nature of the apolar domain and the degree of branching, likely to impact DES component packing and extraction tendencies.

This review aims to establish parallels between type V DESs, sceptically regarded as being of academic curiosity with limited practicality,^34^ to the industrially mature solvent extraction applications. Whilst the inclusion of type V DESs as a subclass of DESs is coherent from a thermodynamic perspective, all physical–chemical properties reviewed indicate that type V DESs are analogous to organic solvents rather than to type I–IV DESs or ionic liquids, as expected based on the precursors. As such, type V DESs should be considered as an *evolution* rather than *revolution* of organic SX phases, which could lower the barriers to industrial implementation if demonstrable improvements are obtained. The notion that the “green” or “sustainable” nature of any DES, including type V, is sufficient to justify its investigation is contradicted by the absence of any studies on the topic. This is not to say that type V DESs are not indeed a greener alternative to conventional phases in SX, rather that there is currently no information to confirm this. Additionally, contributions on the scale-up and continuous application of DESs in SX are encouraged to validate their stability and recyclability under realistic operational conditions.

Although this work exclusively focused on the SX applications of type V DESs, their reported hydrometallurgical applications extend beyond SX to leaching and templates for nanoparticle synthesis. Deep eutectic mixtures of trialkylphosphine oxide with fluorinated β-diketones or decanoic acid were applied (directly or with additives) to the leaching of UO_3_,^168^ the treatment of lithium-ion batteries,^131,171,201^ and REOs.^172^ The last application is particularly interesting as Hanada and co-workers compared the same DES as the organic phase in SX or as leaching media for oxide dissolution, focusing on Fe^3+^, Co^2+^, Nd^3+^, and Dy^3+^.^172^ The DESs exhibited enhanced selectivity for Nd^3+^ when applied as a lixiviant compared to the poor separation afforded with Dy^3+^ and Fe^3+^ during SX, suggesting another avenue for enhancing selectivity in these solvents. Furthermore, the hydrophobic lixiviants were easily regenerated by stripping with aqueous solutions.^131,171,172,201^ Stripping of the eutectic phase after extraction can be bypassed and instead applied to the synthesis of added-value products such as nanoparticles. This was demonstrated for the synthesis of palladium oxide nanoparticles after extraction in the TOPO + thymol mixture,^47^ or the preparation of MgZrO_3_ nanoparticles in thymol + menthol.^202^ Such examples detail the application of type V DESs within integrated hydrometallurgical processes capable of simultaneously combining multiple unit operations, such as leaching and separation, potentiating the economic and sustainability benefits by simplifying the overall process flowsheet.

## Data availability

The data supporting this review are duly referenced and have been included as part of the ESI.†

## Conflicts of interest

There are no conflicts to declare.

## Supplementary Material

GC-027-D5GC00489F-s001
