# Supplementary material for: Examining the potential of type V DESs for the solvent extraction of metal ions
Source: Green Chem. 2025 Mar 25;27(17):4438–63. doi: 10.1039/d5gc00489f (PMC11975223; doi:10.1039/d5gc00489f)
Supplement: GC-027-D5GC00489F-s001 [file GC-027-D5GC00489F-s001.pdf]

Supplementary Information (SI) for Green Chemistry.
This journal is © The Royal Society of Chemistry 2025

# SUPPLEMENTARY INFORMATION

## Examining the Potential of Type V DES for Metal Solvent Extraction

Nicolas Schaeffer,[a*] Inês Vaz,[a] Maísa Saldanha Pinheiro,[a] Felipe Olea,[b] Takafumi Hanada,[c] Sandrine Dourdain,[d] João A.P. Coutinho[a]

[a] CICECO - Aveiro Institute of Materials, Department of Chemistry, University of Aveiro, 3810-193 Aveiro, Portugal

[b] Laboratory of Separation Process Intensification (SPI), Department of Chemical Engineering and Bioprocess, University of Santiago de Chile (USACH)

[c] Department of Applied Chemistry, Graduate School of Technology, Industrial and Social Sciences, Tokushima University, 2-1 Minamijosanjima, Tokushima, Japan

[d] Institut de Chimie Séparative de Marcoule, ICSM, CEA, CNRS, ENSCM, Univ Montpellier, BP 17171, Marcoule, 30207, Bagnols-sur-Cèze, France

* Corresponding email: nicolas.schaeffer@ua.pt

**Table S1.** Experimental solid-liquid equilibrium data (mole fraction of thymol, $x_{Thymol}$, and melting temperature, $T$) measured in this work at atmospheric pressure for binary mixtures of thymol with benzo-12-crown-4 ether. The melting properties (melting temperature, $T_m$ and enthalpy of fusion, $\Delta H_m$) of benzo-12-crown-4 ether were measured by differential scanning calorimetry as $T_m$ = 319.21 K and $\Delta H_m$ = 32.41 kJ $mol^{-1}$.

| $x_{Thymol}$ | $T$ |
|---|---|
| 0.000 | 319.21 |
| 0.137 | 313.57 |
| 0.220 | 307.96 |
| 0.301 | 297.56 |
| 0.409 | 266.80 |
| 0.501 | - |
| 0.600 | - |
| 0.685 | - |
| 0.785 | 304.47 |
| 0.898 | 318.13 |
| 1.000 | 323.50 |

**Table S2.** The activity coefficients at infinite dilution of the HBA ($\ln\gamma_{HBA}$) in HBA-HBD eutectic mixtures at $x_{HBA}$ = 0.5. The labelling of HBDs and HBAs correspond to those in **Figure 3** of the manuscript.

| | | **Hydrogen bond acceptors (HBA)** | | | | | | | | |
|---|---|---|---|---|---|---|---|---|---|---|
| | | **1'** | **2'** | **3'** | **4'** | **5'** | **6'** | **7'** | **8'** | **9'** |
| **Hydrogen Bond Donors (HBD)** | **1** | 0.499 | 0.445 | 0.543 | 0.318 | 0.567 | 0.539 | 0.221 | 0.307 | 0.669 |
| | **2** | 1.033 | 1.089 | 1.049 | 0.621 | 0.948 | 0.970 | 0.899 | 0.955 | 1.224 |
| | **3** | 1.047 | 1.084 | 1.037 | 0.668 | 0.975 | 0.970 | 0.968 | 0.975 | 1.194 |
| | **4** | 0.693 | 0.800 | 0.818 | 0.333 | 0.666 | 0.718 | 0.385 | 0.700 | 1.112 |
| | **5** | 1.420 | 1.321 | 1.688 | 0.619 | 1.264 | 1.598 | 1.073 | 1.755 | 1.718 |
| | **6** | 0.987 | 0.953 | 0.983 | 0.367 | 0.908 | 0.863 | 0.657 | 0.736 | 1.294 |
| | **7** | 0.797 | 0.851 | 0.871 | 0.307 | 0.723 | 0.739 | 0.457 | 0.651 | 1.186 |
| | **8** | 0.859 | 0.745 | 0.788 | 0.600 | 0.936 | 0.773 | 0.796 | 0.884 | 0.902 |
| | **9** | 0.408 | 0.317 | 0.418 | 0.258 | 0.527 | 0.421 | 0.123 | 0.159 | 0.527 |
| | **10** | 0.594 | 0.602 | 0.618 | 0.179 | 0.589 | 0.550 | 0.400 | 0.561 | 0.895 |
| | **11** | 0.756 | 0.771 | 0.793 | 0.395 | 0.757 | 0.729 | 0.564 | 0.737 | 1.014 |
| | **12** | 0.844 | 0.852 | 0.911 | 0.343 | 0.763 | 0.849 | 0.742 | 1.307 | 1.119 |

**Table S3.** List of $\Delta_m H_i$, $\Delta_m S_i$, *and* $T_{m,i}$ for reported components in Type V "deep" and ideal eutectic solvents.

| **Compound** | **$\Delta_m$H (kJ.mol$^{-1}$)** | **$\Delta_m$S (J.mol$^{-1}$K-1)** | **$T_m$ (K)** | **Ref** |
|---|---|---|---|---|
| 1,10-phenanthroline | 15.50 | 39.57 | 391.7 | 1 |
| Pyridine | 8.28 | 35.77 | 231.5 | 2 |
| 2,2'-bipyridine | 20.40 | 59.13 | 345.00 | 3 |
| Lidocaine | 16.40 | 48.14 | 340.7 | 4 |
| Phenol | 11.50 | 36.61 | 314.1 | 5 |
| 2,6-Di-tert-butyl-4-methylphenol | 19.85 | 58.09 | 341.7 | 6 |
| Thymol | 19.65 | 60.74 | 323.5 | 7 |
| Eugenol | 18.72 | 69.54 | 269.2 | 8 |
| Camphor | 6.30 | 13.92 | 452.7 | 9 |
| Menthol | 12.89 | 40.83 | 315.7 | 7 |
| Borneol | 7.30 | 15.16 | 481.6 | 9 |
| Acetylsalicylic Acid | 23.01 | 56.48 | 407.4 | 10 |
| trans-Ferulic Acid | 30.50 | 68.55 | 444.9 | 10 |
| Hydrocinnamic Acid | 16.30 | 50.65 | 321.8 | 11 |
| Salicylic Acid | 24.45 | 56.72 | 431.1 | 10 |
| Syringic Acid | 33.70 | 70.16 | 480.3 | 10 |
| Trioctylphosphine oxide | 58.02 | 178.03 | 325.9 | 11 |
| Triphenylphosphine oxide | 24.22 | 56.08 | 431.9 | 12 |
| N,N′-dihexylthiourea | 20 | 62.75 | 318.7 | 13 |
| Thenoyltrifluoroacetone | 12.05 | 37.89 | 318.0 | 14 |
| Benzoyltrifluoroacetone | 19.17 | 61.25 | 313.0 | 14 |
| 18-Crown-6-ether | 40.0 | 128.08 | 312.3 | 15 |
| Dibenzo-18-Crown-6-ether | 56.0 | 128.53 | 435.7 | 15 |
| Dibenzo-24-Crown-8-ether | 53.7 | 143.62 | 373.9 | 15 |
| Dibenzo-30-Crown-10-ether | 86.5 | 229.08 | 377.6 | 15 |
| Malonic Acid | 23.1 | 56.69 | 407.5 | 16 |
| Levulinic Acid | 9.22 | 30.11 | 306.2 | 17 |
| Capric Acid | 27.50 | 90.22 | 304.8 | 7 |
| Decanol | 33.7 | 120.36 | 280 | 18 |
| Tertbutanol | 5.6 | 18.75 | 298.7 | 19 |
| perfluoro tert-butanol | 8.2 | 32.58 | 251.7 | 19 |

**Table S4.** Reported viscosity and density of non-ionic hydrophobic eutectic solvents at 298 K as a function of composition and constituents (HTTA – thenoyltrifluoroacetone; HBTA – benzoyl trifluoroacetone; TOPO – trioctylphosphine oxide; TBP – tributylphosphate; D2EHPA – di(2-ethylhexyl)phosphoric acid).

| System (Component 1 / Component 2) | $x_{Component1}$ | Viscosity | Density | Ref |
|---|---|---|---|---|
| HBTA / N, N-bis (2-ethylhexyl) acetamide | 0.50 | 16.82 | 0.976 | 20 |
| HBTA / TBP | 0.50 | 9.76 | 1.07 | 20 |
| HBTA / TOPO | 0.50 | 11.2 | 0.984 | 20 |
| HTTA / TBP | 0.50 | 5.04 | 1.13 | 21 |
| HTTA / N, N-dimethylbenzamide | 0.50 | 8.13 | 1.25 | 21 |
| HTTA / TOPO | 0.67 | 23.15 | 1.1 | 21 |
| HBTA / TOPO | 0.67 | 14.44 | 1.06 | 21 |
| HTTA / TOPO | 0.67 | 28.1 | 1.1 | 14 |
| HBTA / TOPO | 0.67 | 13.2 | 1.05 | 14 |
| HTTA / Triphenyl phosphate | 0.67 | 11.6 | 1.32 | 14 |
| HTTA / DDA | 0.67 | 18.06 | 1.06 | 22 |
| HTTA / DDA | 0.50 | 16.24 | 1.05 | 22 |
| HTTA / DDA | 0.33 | 12.85 | 0.97 | 22 |
| TOPO / Decanol | 0.33 | 30.3 | 0.85 | 23 |
| TOPO / Thymol | 0.50 | 69.93 | 0.898 | 11 |
| TOPO / Decanoic acid | 0.50 | 44.11 | 0.881 | 11 |
| TOPO / Phenol | 0.33 | 12.38 | 0.933 | 24 |
| TOPO / Phenol | 0.50 | 43.0 | 0.907 | 24 |
| TOPO / Dodecanol | 0.33 | 27.52 | 0.849 | 25 |
| TOPO / Dodecanol | 0.25 | 25.77 | 0.846 | 25 |
| TOPO / Dodecanol | 0.20 | 23.55 | 0.843 | 25 |
| TOPO / Dodecanol | 0.17 | 23.53 | 0.842 | 25 |
| TOPO / Dodecanol | 0.14 | 21.19 | 0.841 | 25 |
| TOPO / Malonic acid | 0.55 | - | 0.941 | 26 |
| TOPO / Levulinic acid | 0.40 | - | 0.940 | 26 |
| Hydrocinamic acid / Decanoic acid | 0.50 | 11.29 | 0.978 | 11 |
| Decanoic acid / Dodecanoic acid/ | 0.67 | 10.76 | 0.894 | 27 |

| | | | | |
|---|---|---|---|---|
| Decanoic acid / Lidocaine | 0.67 | 237.5 | 0.958 | 28 |
| Decanoic acid / Thymol | 0.50 | 12.16 | 0.930 | 7 |
| Decanoic acid / Menthol | 0.40 | 18.85 | 0.897 | 7 |
| Thymol / Phenanthroline | 0.80 | 75.31 | 1.024 | 29 |
| Thymol / 2-methyl-2,4-pentanediol | 0.67 | 32.69 | 0.959 | 30 |
| Menthol / 2-methyl-2,4-pentanediol | 0.67 | 68.39 | 0.901 | 30 |
| Thymol / Menthol | 0.50 | 53.14 | 0.937 | 8 |
| Menthol / Camphor | 0.50 | 16.42 | 0.924 | 8 |
| Menthol / Borneol | 0.70 | 110.4 | 0.915 | 8 |
| Thymol / Camphor | 0.50 | 20.8 | 0.967 | 8 |
| Menthol / D2EHPA | 0.50 | 31.28 | 0.947 | 31 |
| N,N-diisooctylacetamide / Decanol | 0.50 | 27.05 | 0.866 | 32 |

**Table S5.** Experimentally reported Kamlet-Taft parameters of non-ionic eutectic solvents.

| Component 1 | Component 2 | $x_{Component1}$ | $\alpha$ | $\beta$ | $\pi$ | Ref |
|---|---|---|---|---|---|---|
| Menthol | Acetic acid | 0.50 | 1.64 | 0.6 | 0.53 | 33 |
| | Levulinic acid | 0.50 | 1.56 | 0.58 | 0.66 | |
| | Octanoic acid | 0.50 | 1.77 | 0.5 | 0.41 | |
| | Dodecanoic acid | 0.33 | 1.79 | 0.57 | 0.37 | |
| Menthol | Octanoic acid | 0.43 | 0.85 | 0.43 | 0.39 | 7 |
| | Decanoic acid | 0.57 | 0.84 | 0.45 | 0.35 | |
| | Dodecanoic acid | 0.68 | 0.79 | 0.54 | 0.37 | |
| | Tetradecanoic acid | 0.78 | 0.75 | 0.5 | 0.38 | |
| | Hexadecanoic acid | 0.85 | 0.71 | 0.57 | 0.38 | |
| | Octadecanoic acid | 0.91 | 0.68 | 0.64 | 0.38 | |
| Thymol | Octanoic acid | 0.29 | 1.1 | 0.05 | 0.67 | |
| | Decanoic acid | 0.43 | 1.11 | 0.05 | 0.71 | |
| | Dodecanoic acid | 0.55 | 1.05 | 0.02 | 0.75 | |
| | Tetradecanoic acid | 0.67 | 1.13 | 0.02 | 0.84 | |
| | Hexadecanoic acid | 0.76 | 1.11 | 0.01 | 0.87 | |
| | Octadecanoic acid | 0.84 | 1.1 | 0.05 | 0.94 | |
| Menthol | Thymol | 0.10 | 1 | 0.15 | 0.97 | 8 |
| | | 0.20 | 0.97 | 0.18 | 0.9 | |
| | | 0.30 | 0.92 | 0.22 | 0.84 | |
| | | 0.40 | 0.88 | 0.23 | 0.83 | |
| | | 0.50 | 0.84 | 0.28 | 0.77 | |
| | | 0.60 | 0.79 | 0.32 | 0.72 | |
| | | 0.70 | 0.7 | 0.36 | 0.67 | |
| | | 0.80 | 0.64 | 0.48 | 0.59 | |
| | | 0.90 | 0.56 | 0.53 | 0.53 | |
| Menthol | Camphor | 0.50 | 0.41 | 0.61 | 0.52 | |
| | Borneol | 0.70 | 0.53 | 0.63 | 0.43 | |
| | Sobrerol | 0.95 | 0.52 | 0.68 | 0.43 | |
| Thymol | Camphor | 0.30 | 0.68 | 0.45 | 0.59 | |
| | Camphor | 0.40 | 0.71 | 0.41 | 0.62 | |

| | | | | |
|---|---|---|---|---|
| Camphor | 0.50 | 0.82 | 0.34 | 0.69 |
| Camphor | 0.60 | 0.88 | 0.26 | 0.77 |
| Camphor | 0.70 | 0.94 | 0.23 | 0.85 |
| Camphor | 0.80 | 0.97 | 0.18 | 0.93 |
| Camphor | 0.90 | 1.01 | 0.17 | 0.98 |
| Borneol | 0.50 | 0.87 | 0.27 | 0.78 |
| Sobrerol | 0.70 | 0.99 | 0.11 | 0.94 |

**REFERENCES**

1. Chirico, R. D., Kazakov, A. F. & Steele, W. V. Thermodynamic properties of three-ring aza-aromatics. 2. Experimental results for 1,10-phenanthroline, phenanthridine, and 7,8-benzoquinoline, and mutual validation of experiments and computational methods. *J Chem Thermodyn* **42**, 581–590 (2010).

2. Domalski, E. S. & Hearing, E. D. Heat Capacities and Entropies of Organic Compounds in the Condensed Phase. Volume III. *J Phys Chem Ref Data* **25**, 1 (2009).

3. Lipkind, D., Hanshaw, W. & Chickos, J. S. Hypothetical thermodynamic properties. Subcooled vaporization enthalpies and vapor pressures of polyaromatic heterocycles and related compounds. *J Chem Eng Data* **54**, 2930–2943 (2009).

4. Lazerges, M., Rietveld, I. B., Corvis, Y., Céolin, R. & Espeau, P. Thermodynamic studies of mixtures for topical anesthesia: Lidocaine–salol binary phase diagram. *Thermochim Acta* **497**, 124–128 (2010).

5. Andon, R. J. L., Counsell, J. F., Herington, E. F. G. & Martin, J. F. Thermodynamic properties of organic oxygen compounds. Part 7.—Calorimetric study of phenol from 12 to 330°K. *Trans Faraday Soc* **59**, 830–835 (1963).

6. Verevkin, S. P. Thermochemistry of phenols: buttress effects in sterically hindered phenols. *J Chem Thermodyn* **31**, 1397–1416 (1999).

7. Martins, M. A. R. *et al.* Tunable Hydrophobic Eutectic Solvents Based on Terpenes and Monocarboxylic Acids. *ACS Sustain Chem Eng* **6**, 8836–8846 (2018).

8. Martins, M. A. R. *et al.* Greener Terpene-Terpene Eutectic Mixtures as Hydrophobic Solvents. *ACS Sustain Chem Eng* **7**, 17414–17423 (2019).

9. Chandra, G. & Murthy, S. S. N. Dielectric and thermodynamic study of camphor and borneol enantiomers and their binary systems. *Thermochim Acta* **666**, 241–252 (2018).

10. Su, C. S. & Chen, Y. P. Correlation for the solubilities of pharmaceutical compounds in supercritical carbon dioxide. *Fluid Phase Equilib* **254**, 167–173 (2007).

11. Schaeffer, N. *et al.* Non-ionic hydrophobic eutectics-versatile solvents for tailored metal separation and valorisation. *Green Chem* **22**, 2810–2820 (2020).

12. Hulnink, J., van Miltenburg, K., Oonk, H. A. J., Schuljff, A. & Groen, P. Thermodynamic Functions and Vapor Pressures of Triphenylphosphine Oxide and 1,4-Bis(diphenylphosphino)butane Near the Melting Point. *J Chem Eng Data* **34**, 99–100 (1989).

13. van den Bruinhorst, A. *et al.* Hydrophobic eutectic mixtures as volatile fatty acid extractants. *Sep Purif Technol* **216**, 147–157 (2019).

14. Hanada, T. & Goto, M. Synergistic Deep Eutectic Solvents for Lithium Extraction. *ACS Sustain Chem Eng* **9**, 2152–2160 (2021).

15. Sánchez-Bulás, T., Cruz-Vásquez, O., Hernández-Obregón, J. & Rojas, A. Enthalpies of fusion, vaporisation and sublimation of crown ethers determined by thermogravimetry and differential scanning calorimetry. *Thermochim Acta* **650**, 123–133 (2017).

16. Hansen, A. R. & Beyer, K. D. Experimentally Determined Thermochemical Properties of the Malonic Acid/Water System: Implications for Atmospheric Aerosols. *J Phys Chem A* **108**, 3457–3466 (2004).

17. Acree, W. E. Thermodynamic properties of organic compounds: enthalpy of fusion and melting point temperature compilation. *Thermochim Acta* **189**, 37–56 (1991).

18. Van Miltenburg, J. C., Gabrielová, H. & Růžička, K. Heat capacities and derived thermodynamic functions of 1-hexanol, 1-heptanol, 1-octanol, and 1-decanol between 5 K and 390 K. *J Chem Eng Data* **48**, 1323–1331 (2003).

19. Vaz, I. C. M. *et al.* The path towards type V deep eutectic solvents: inductive effects and steric hindrance in the system tert-butanol + perfluoro tert-butanol. *Phys Chem Chem Phys* **25**, 11227–11236 (2023).

20. Zhang, L., Li, J., Ji, L. & Li, L. Separation of lithium from alkaline solutions with hydrophobic deep eutectic solvents based on β-diketone. *J Mol Liq* **344**, 117729 (2021).

21. Luo, H. *et al.* Selective recovery of lithium from mother liquor of Li2CO3 by synergistic hydrophobic deep eutectic solvents: Performance and mechanistic insight. *Sep Purif Technol* **313**, 123353 (2023).

22. Chen, J. *et al.* A novel ternary hydrophobic deep eutectic solvent over a wide pH range for lithium recovery. *J Hazard Mater* **480**, 136398 (2024).

23. Yu, G. *et al.* Recovery of rare earth metal oxides from NdFeB magnet leachate by hydrophobic deep eutectic solvent extraction, oxalate stripping and calcination. *Hydrometallurgy* **223**, 106209 (2024).

24. Gilmore, M. *et al.* Hydrophobic Deep Eutectic Solvents Incorporating Trioctylphosphine Oxide: Advanced Liquid Extractants. *ACS Sustain Chem Eng* **6**, 17323–17332 (2018).

25. Ni, S. *et al.* A cleaner strategy for comprehensive recovery of waste SmCo magnets based on deep eutectic solvents. *Chem Eng J* **412**, 128602 (2021).

26. Byrne, E. L. *et al.* Hydrophobic functional liquids based on trioctylphosphine oxide (TOPO) and carboxylic acids. *Phys Chem Chem Phys* **22**, 24744–24763 (2020).

27. Florindo, C., Romero, L., Rintoul, I., Branco, L. C. & Marrucho, I. M. From Phase Change Materials to Green Solvents: Hydrophobic Low Viscous Fatty Acid−Based Deep Eutectic Solvents. A*CS Sustain Chem. Eng.* **6**, 3888–3895 (2018).

28. Bica, K., Shamshina, J., Hough, W. L., Macfarlane, D. R. & Rogers, R. D. Liquid forms of pharmaceutical co-crystals: exploring the boundaries of salt formation. *Chem Comm* **47**, 2267 (2011).

29. Crema, A. P. S. *et al.* New family of Type V eutectic solvents based on 1,10-phenanthroline and their application in metal extraction. *Hydrometallurgy* **215**, 105971 (2023).

30. Almustafa, G. *et al.* Boron extraction from aqueous medium using novel hydrophobic deep eutectic solvents. *Chem Eng J* **395**, 125173 (2020).

31. Zinov'eva, I. V., Kozhevnikova, A. V., Milevskii, N. A., Zakhodyaeva, Y. A. & Voshkin, A. A. Extraction of Cu(II), Ni(II), and Al(III) with the Deep Eutectic Solvent D2EHPA/Menthol. *Theor Found Chem Eng* **56**, 221–229 (2022).

32. Zhu, K., Wei, Q., Li, H. & Ren, X. Recovery of Titanium from Ilmenite HCl Leachate Using a Hydrophobic Deep Eutectic Solvent. *ACS Sustain Chem Eng* **10**, 2125–2135 (2022).

33. Florindo, C., McIntosh, A. J. S., Welton, T., Branco, L. C. & Marrucho, I. M. A closer look into deep eutectic solvents: exploring intermolecular interactions using solvatochromic probes. *Phys Chem Chem Phys* **20**, 206–213 (2017).
